# Surfactant-free syntheses and pair distribution function analysis of osmium nanoparticles

**DOI:** 10.3762/bjnano.13.17

**Published:** 2022-02-16

**Authors:** Mikkel Juelsholt, Jonathan Quinson, Emil T S Kjær, Baiyu Wang, Rebecca Pittkowski, Susan R Cooper, Tiffany L Kinnibrugh, Søren B Simonsen, Luise Theil Kuhn, María Escudero-Escribano, Kirsten M Ø Jensen

**Affiliations:** 1 Department of Chemistry, University of Copenhagen, 5 Universitetsparken, Copenhagen, 2100, Denmark; 2 X-ray Science Division, Advanced Photon Source, Argonne National Laboratory, Argonne, 9700 S Cass Ave, Lemont, IL 60439, USA; 3 Department of Energy Conversion and Storage, Technical University of Denmark, Fysikvej Bldg. 310, Lyngby, DK-2800 Kgs., Denmark

**Keywords:** metal nanoparticles, osmium, pair distribution function, surfactant-free synthesis

## Abstract

A surfactant-free synthesis of precious metal nanoparticles (NPs) performed in alkaline low-boiling-point solvents has been recently reported. Monoalcohols are here investigated as solvents and reducing agents to obtain colloidal Os nanoparticles by using low-temperature (<100 °C) surfactant-free syntheses. The effect of the precursor (OsCl_3_ or H_2_OsCl_6_), precursor concentration (up to 100 mM), solvent (methanol or ethanol), presence or absence of a base (NaOH), and addition of water (0 to 100 vol %) on the resulting nanomaterials is discussed. It is found that no base is required to obtain Os nanoparticles as opposed to the case of Pt or Ir NPs. The robustness of the synthesis for a precursor concentration up to 100 mM allows for the performance of X-ray total scattering with pair distribution function (PDF) analysis, which shows that 1–2 nm hexagonal close packed (hcp) NPs are formed from chain-like [OsO*_x_*Cl*_y_*] complexes.

## Findings

Precious metals are limited resources, yet fundamental for a range of applications, such as in medicine or catalysis [1–3]. There are relatively few reports on osmium (Os) compared to other precious metals [4–5], see Figure S1 in Supporting Information File 1, partly due to Os scarcity and because a highly toxic OsO_4_ compound can be easily formed. However, Os and Os-based complexes and nanomaterials have been reported [6–9] and studied for their catalytic, optical, and medical properties [10–17] in experimental and theoretical work [18].

Common wet chemical syntheses require surfactants, viscous solvents, or shape-directing agents [5,17,19–21] that act as ligands to stabilize colloidal nanoparticles (NPs). These additives can bring impurities, be toxic, and add cost to the synthesis. Surfactant-free syntheses, therefore, bear promising features not only for fundamental research but also for industrial scale production [22–24]. A surfactant-free synthesis method to produce Pt, Ir, Ru, or bimetallic NPs has recently been reported [22]. It only requires a monoalcohol as solvent and reducing agent [25], a base, and a metal precursor to obtain size-controlled NPs [26–27]. This approach leads to catalysts that are more active than those prepared, for example, in polyols [28–29]. Here we investigate whether this simple synthetic approach using monoalcohols is suitable for the synthesis of Os NPs.

Here, the synthesis of Os NPs is easily performed as described in detail in the Materials and Methods section in Supporting Information File 1. The precursors used were solutions of OsCl_3_ and H_2_OsCl_6_ and the syntheses were performed in closed containers made of polypropylene, which were heated to 90 °C for 6 h using an oil bath. As opposed to a classical reflux setup using glassware [22] or to the use of microwaves [30], visible or UV light [31], this allowed for a rapid screening of a number of experimental parameters for long synthesis times. Studies of NP synthesis have made it clear that a number of seemingly simple experimental parameters can play a significant role in nanomaterial synthesis [5,32]. We therefore screened the influence of several experimental parameters across a large parametric space by investigating the influence of the precursors: OsCl_3_ and H_2_OsCl_6_, the nature of the solvent/reducing agent: methanol and ethanol, the absence or presence of a base (NaOH), and the effect of adding water at: 0, 10, 25, 50, 66, 75 or 100 vol %. Table S1, Supporting Information File 1, gives an overview of the different parameters investigated for this parametric study and Figures S2–S21, Supporting Information File 1, gather transmission electron microscopy (TEM) micrographs and pictures of the materials synthesized.

**Figure 1:**
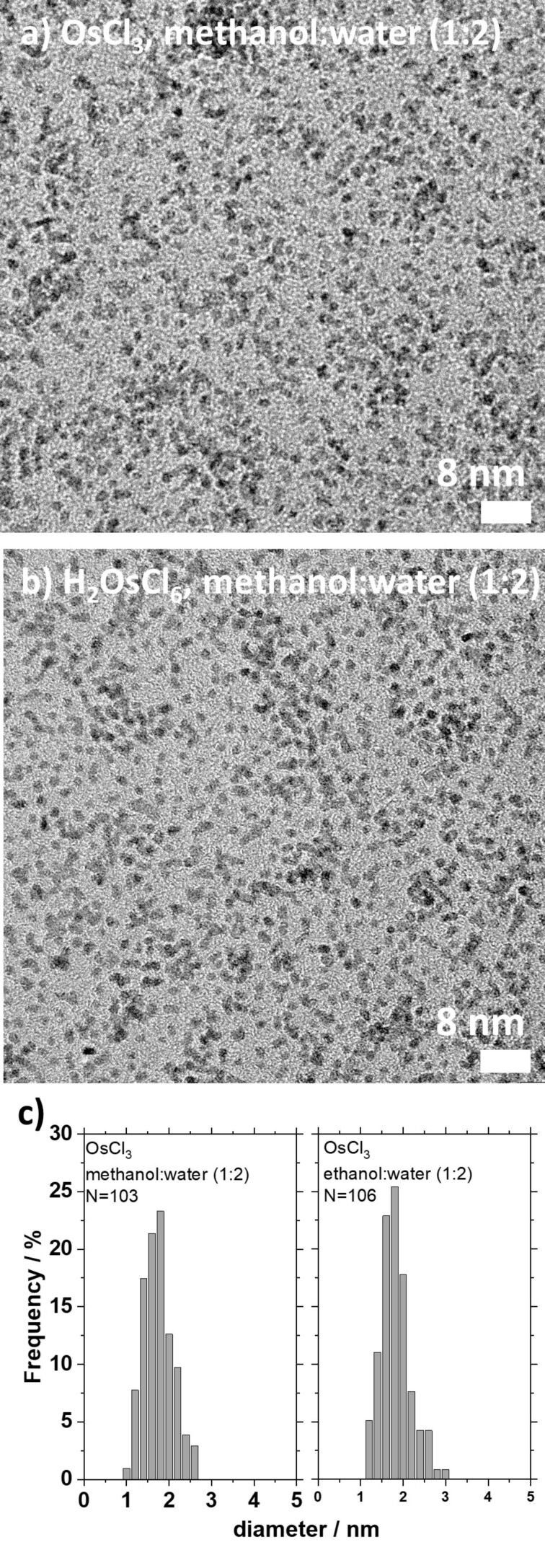
TEM micrographs of Os NPs obtained using water (66 vol %) and methanol (33 vol %), no base, and 100 mM of (a) OsCl_3_ and (b) H_2_OsCl_6_ as precursors after a one-week reaction at 85 °C in nuclear magnetic resonance (NMR) tubes (volume approx. 0.2 mL). The size analysis suggests that the NPs are (a) 1.6 ± 0.4 nm and (b) 1.7 ± 0.3 nm. The size distribution is presented in (c) from the analysis of 103 and 106 NPs, respectively.

Our results clearly show that small Os NPs can be easily obtained from this simple synthesis method. For example, TEM images of Os NPs from two different syntheses are shown in Figure 1. In both cases, the synthesis results in Os NPs of approx. 1–2 nm. When further comparing the results from the large parameter space (Figures S2–S21, Supporting Information File 1), it is clear that the synthesis conditions, in fact, have little influence on the resulting size of the Os NPs, which in all cases is 1–2 nm. The synthesis product does not appear to be affected by the choice of the precursor material, as seen when comparing products obtained from OsCl_3_ and H_2_OsCl_6_ (e.g., Figures S6 and S7, Supporting Information File 1). As opposed to the case of Pt NP synthesis, the Os NP size is also not affected by the alcohol used as reductant, as similar NP sizes are obtained when considering the TEM results (comparing Figure S6 and Figure S8, Supporting Information File 1). This result is confirmed by size analysis using small angle X-ray scattering (SAXS) analysis as presented in Figure S22 and Table S2, Supporting Information File 1.

However, our experiment showed that either methanol or ethanol is needed for the reaction to proceed playing the role of reducing agents, as no product is formed when the synthesis is performed in 100% water. Furthermore, the water content in the solvent affects the general morphology of the product: While dispersed NPs are obtained in pure ethanol or methanol, adding water leads to the formation of network-like structures as reported in Figures S2–S18 (Supporting Information File 1) although the exact nature of this network is not yet established. The networks formed in the presence of water are not observed in the synthesis of Pt, Ir, or Ru NPs [22,26,29]. Our data show that, as opposed to Pt and Ir NP syntheses, Os NP synthesis proceeds without the need for a base (Figures S18, S20, S21 of Supporting Information File 1).

To develop a simple and relatively inexpensive synthesis of Os NPs requiring only few chemicals, it was concluded that OsCl_3_, no base, and a relatively high-water content (around 60–75 vol %) using methanol as the reducing agent were favourable conditions to obtain Os NPs for further studies. Transmission electron microscopy images from samples synthesized with these parameter sets are shown in Figure 1.

The small size of the nanoparticles challenges conventional structural characterization methods such as X-ray diffraction analysis (XRD). Here, we turned to X-ray total scattering and pair distribution function (PDF) analyses to investigate the atomic structure of the Os NPs [33]. The pair distribution function is now widely used for nanomaterial characterization, as it allows to obtain atomic structure information from materials showing no long range order [34].

**Figure 2:**
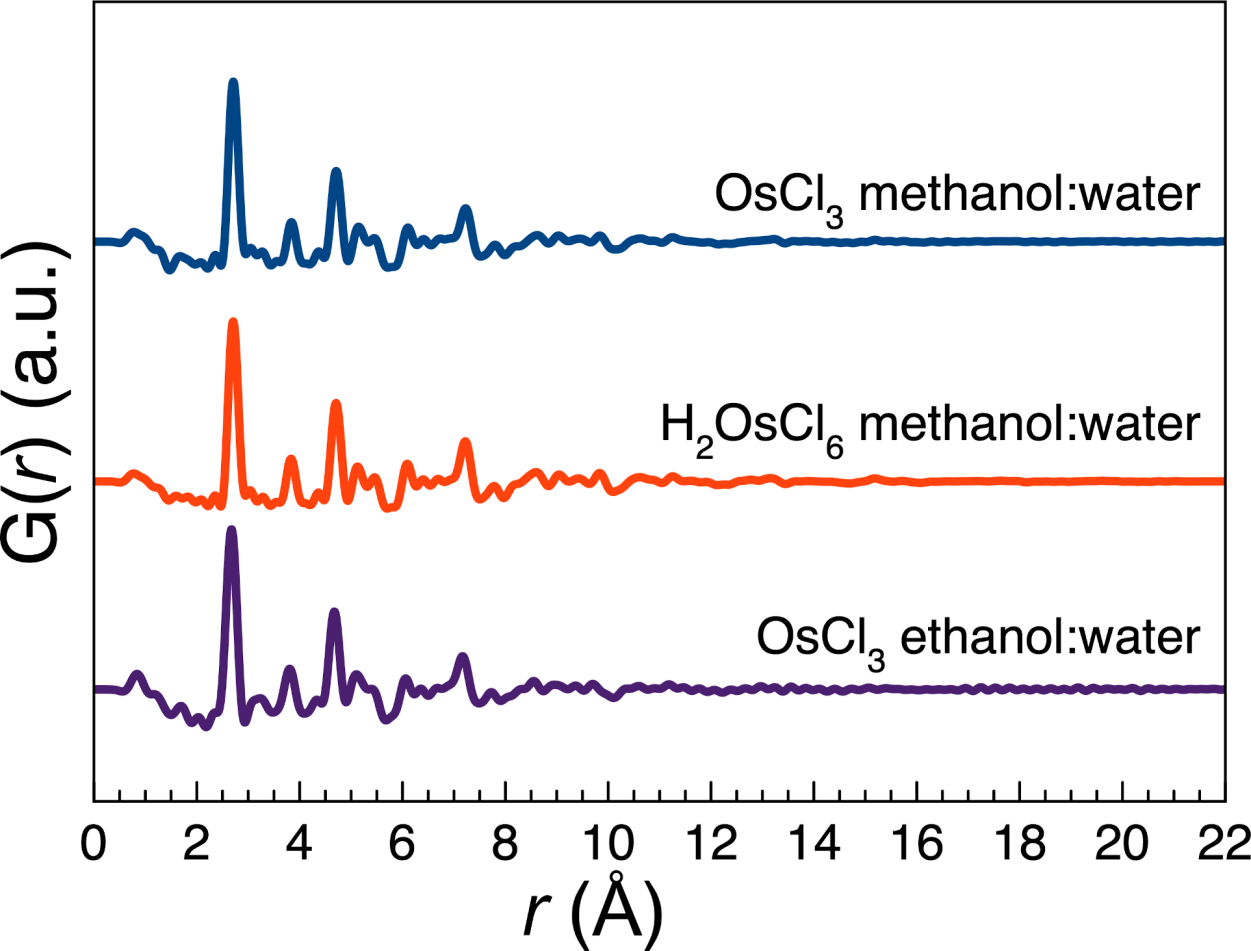
PDFs obtained from three different syntheses of Os NPs in 1:2 alcohol/water ratios and for different precursors as indicated. The PDFs can be described using a hcp model.

The total scattering signal is shown in Supporting Information File 1, Figure S25 and the resulting PDFs in Figure 2. The PDFs of the three samples are more or less identical, which is in line with the parameter study where these parameters do not influence the formed NPs. From the extent of features in the PDFs, it can be concluded that the NPs are all smaller than 2 nm with an average crystallite size between 1 and 2 nm, which is in agreement with TEM and SAXS characterisation.

On the nanoscale, metallic NPs can have different structures than those of bulk metals, and icosahedral or decahedral motifs are often observed for metals with normally close-packed structures [35]. By using the atomic simulation environment (ASE) module [36] and the cluster-mining approach developed by Banerjee et al. [35] we screened a large number of metal NP clusters, including hexagonal close packed (hcp), face-centred cubic (fcc), and body-centred cubic (bcc), and also structures such as icosahedrons and decahedrons.

**Figure 3:**
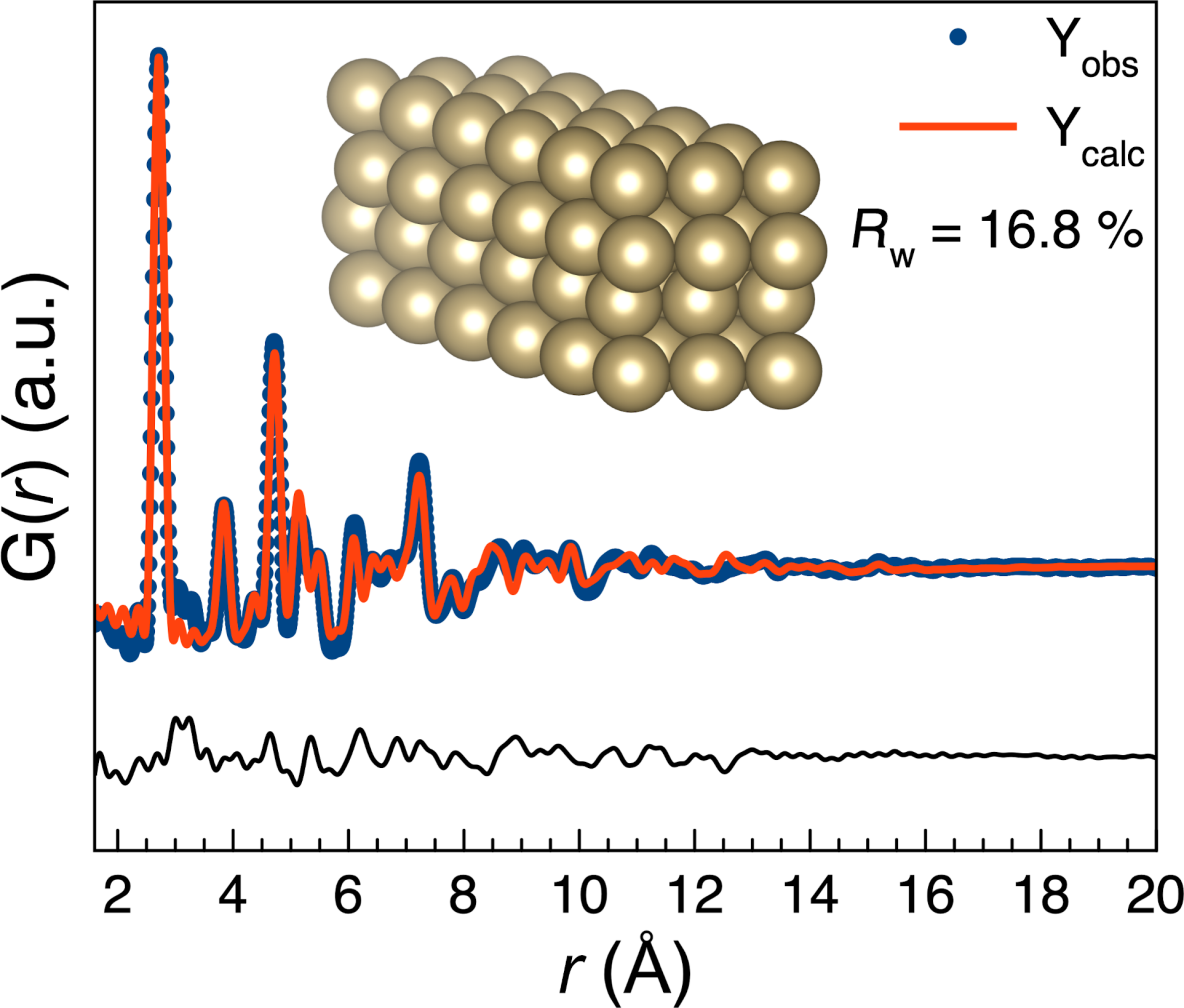
Fit of a hcp cluster (seen in the insert) to the PDF obtained from the Os NPs formed in methanol/water from OsCl_3_.

The synthesised Os NPs are best described using a small hcp cluster as seen in Figure 3 and Figures S26–S31, which agrees with the bulk hcp Os structure. As seen in Figure S39, Supporting Information File 1, the PDFs from the Os NPs synthesised from OsCl_3_ in ethanol/water show a small peak corresponding to an interatomic distance at 3.2 Å. A small indication of the same peak can be seen as a shoulder for the two samples synthesized in methanol. The distance is not described in the hcp model. We considered whether this peak could be originated from a ligand binding to the surface of the NPs. However, 3.2 Å is significantly longer than Os–O, Os–C, and Os–Cl bond distances which are approx. 1.8, 1.9, and 2.3 Å, respectively. However, this peak at 3.2 Å could correspond to an Os–Os distance in a [Os_2_Cl_2_] complex which may be present in the solution, see Supporting Information File 1. A PDF from NPs synthesised from H_2_OsCl_6_ in ethanol/water was also obtained and is shown in Figure S40, Supporting Information File 1. As discussed in Supporting Information File 1, this PDF shows that NPs synthesised from H_2_OsCl_6_ in ethanol/water got oxidised and so while the NPs remain mainly non-oxidised in solution, they can get oxidised over time or upon drying to form OsO_4_.

**Figure 4:**
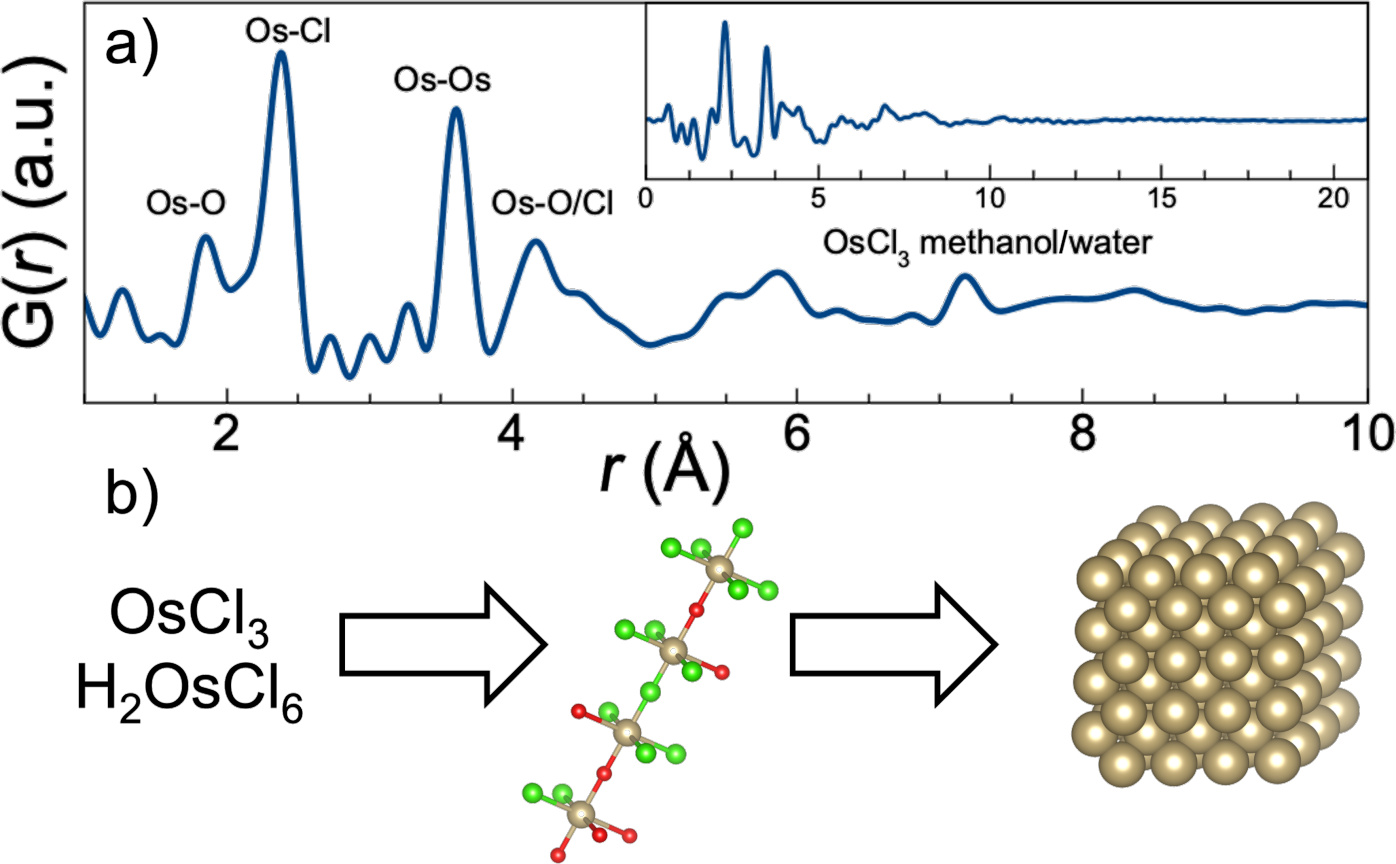
(a) Measured PDF of OsCl_3_ in methanol/water. The insert shows the same PDF plotted to 21 Å. (b) Overall formation mechanism of the hcp Os NPs. The Os chloride precursor reacts with the alcohol/water mixture to form chain-like structures of [OsO*_x_*Cl*_y_*]-octahedra, which after a long incubation time form Os NPs. Os, Cl, and O atoms are shown in grey, green, and red, respectively.

Total scattering experiments on the precursor solution were also performed, see Figure 4 and Figures S41–S45, Supporting Information File 1. The resulting PDFs are very similar to the PDFs of crystalline OsOCl compounds (e.g., OsOCl_2_ and (NH_4_)_4_(OsOCl_10_)), which form chain-like structures. Both structures are shown in Figures S43 and S44, Supporting Information File 1. This indicates that the precursors react with the solvent and form a network of chain-like structures of [OsO*_y_*Cl*_x_*]-octahedra, as illustrated in Figure 4. Note that O atoms could be from either a methoxy or ethoxy groups, although PDF does not allow us to firmly draw conclusions at this stage. The network is quite stable and even after 6 h at 85 °C, minimal changes in the PDF occur for this high concentration of precursor (100 mM) syntheses, see Figure S46, Supporting Information File 1. This stresses the need for longer syntheses times to initiate the breakdown of the precursor at a high precursor concentration. This observation is in line with recent reports on the formation mechanism involving chain-like structures made for the synthesis of Pt NPs [33].

## Conclusion

In conclusion, Os NPs with a hcp crystal structure and a size of approx. 1–2 nm are synthesized in methanol, ethanol, and water mixtures of OsCl_3_ or H_2_OsCl_6_ precursors, without the need for surfactants. Despite the absence of surfactants, the small-sized NPs are stable, in agreement with previous surfactant-free Os NP syntheses [37]. This can be attributed to chloride stabilization and/or stabilization by oxidation products of the monoalcohols [25]. Here, as opposed to the synthesis of Pt, Ir, Ru, or Pd NPs by a similar approach, no base is required. In addition, a synthesis method using methanol/water in the volume ratio 1:2 with OsCl_3_ as a precursor was considered optimal. The results presented show that size control of Os NPs is a challenging task and even at a high precursor concentration of up to 100 mM, small-sized NPs (1–2 nm in diameter) are obtained. X-ray total scattering measurements with PDF analysis show that the samples have the hcp structure, and it is shown that Os NPs are formed from [OsO*_x_*Cl*_y_*] complexes. This study and the versatile synthesis introduced provide a suitable platform to inspire future studies on the formation mechanism of Os-based nanomaterials in which their properties are further explored.

## Supporting Information

Figure S1: Literature survey on precious metals. Figures S2–S21: TEM characterization and pictures of the nanomaterials obtained. Figure S22: SAXS characterization of the nanomaterials obtained. Figure S23: TEM characterization of the nanomaterials obtained. Figure S24: XRD characterization of the nanomaterials obtained. Figures S25–42: PDF characterization of the nanomaterials obtained. Figures S43–45: Crystal structures and models of the different clusters and complexes. Figure S46: PDF characterization of the nanomaterials obtained. Table S1: Overview of the parameters studied. Table S2: Fit parameters for SAXS. Tables S3–S13: Refined parameters for PDF.

File 1 Additional experimental information.
