# Supplementary material for: Surfactant-free syntheses and pair distribution function analysis of osmium nanoparticles
Source: Beilstein J Nanotechnol. 2022 Feb 16;13:230–5. doi: 10.3762/bjnano.13.17 (PMC8895034; doi:10.3762/bjnano.13.17)
Supplement: File 1 — Additional experimental information. [file Beilstein_J_Nanotechnol-13-230-s001.pdf]

BEILSTEIN JOURNAL OF NANOTECHNOLOGY

Supporting Information

for

Surfactant-free syntheses and pair distribution function analysis of osmium nanoparticles

Mikkel Juelsholt, Jonathan Quinson, Emil T. S. Kjær, Baiyu Wang, Rebecca Pittkowski, Susan R. Cooper, Tiffany L. Kinnibrugh, Søren B. Simonsen, Luise Theil Kuhn, María Escudero-Escribano and Kirsten M. Ø. Jensen

Beilstein J. Nanotechnol. **2022,** *13,* 230–235. doi:10.3762/bjnano.13.17

Additional experimental information

License and Terms: This is a supporting information file under the terms of the Creative Commons Attribution License (https://creativecommons.org/licenses/by/4.0). Please note that the reuse, redistribution and reproduction in particular requires that the author(s) and source are credited and that individual graphics may be subject to special legal provisions.
The license is subject to the *Beilstein Journal of Nanotechnology* terms and conditions: (https://www.beilstein-journals.org/bjnano/terms)

Materials and Methods

Chemicals

The chemicals used in the experiments were: $OsCl_3$ (Premion®, 99.99% metals basis), H_2OsCl_6 (Premion®, 99.95% metals basis), NaOH (98%, Alfa Aesar), methanol (≥99.9%, HiPerSolv Chromanorm®, VWR), ethanol (≥99.5%, absolute, VWR) and water (Milli-Q, Millipore, resistivity >18.2 MΩ·cm).

Osmium nanoparticle synthetic procedures

Parametric study

The precursors for the Os nanomaterial syntheses were obtained from 2.5 mM solutions of $OsCl_3$ or H_2OsCl_6. The solvents used were methanol, ethanol, or their mixture with water. Different water/alcohol ratios were used, as listed in Table S1. Here, the vol % corresponds to the ratios before volume contraction upon mixing. The syntheses were performed in the absence or presence of base as indicated, but in most cases 50 mM NaOH was used. The total volume of the precursor solution used was 2 mL (evaluated as the sum of the volume of water and alcohol before volume contraction). The mixtures were placed in sealed polypropylene tubes (15 mL centrifuge tubes, VWR, melting point of 160 °C) and placed in an oil bath, preheated at 90 °C. The syntheses ran for 6 h to obtain Os nanomaterials.

Samples for PDF analysis

In a second approach, for the purpose of X-ray scattering with PDF analysis which requires a high concentration of scattering material, the samples were prepared in 3 mm NMR tubes (Wildmad, 3 mm outer diameter, 0.27 mm wall thickness, type 1 class A) with a stirrer bar (Fisherbrand PTFE stirrer bar, 8 ×1.5 mm). Here, the precursor concentration was 100 mM ($OsCl_3$ or H_2OsCl_6), and the samples were prepared in 0.2 mL of methanol or ethanol and water mixture in a volume ratio of alcohol/water 1:2. Identical samples, but without $OsCl_3$ or H_2OsCl_6, were used for background measurements. The samples (with and without a metal precursor) were left to react at 85 °C for several hours for in situ measurements or for a week (with precursor) under stirring, as indicated. After removing the stirrer bar, the sealed NMR tubes were shipped from Copenhagen, Denmark to 11-ID-B beamline of the Advanced Photon Source (APS) at the Argonne National Lab, USA, for measurements. TEM analysis confirms the formation of 1–2 nm NPs, see Figure 1 and Figure S23, (i.e., the materials are comparable to those obtained in syntheses done in larger volumes and lower Os concentrations, see Figures S2–S18).

Characterisation

Transmission electron microscopy

A few drops of the as prepared nanomaterials were dropped on TEM grids (Ni, Quantifoil) prior to imaging with a Jeol 2100 transmission electron microscope. For each sample, images were recorded in a least three randomly selected areas with at least three different magnifications. The NPs size was estimated by measuring the diameter of at least 100 NPs using the ImageJ software.

X-ray diffraction

X-ray powder diffraction measurements were performed on a Bruker D8 diffractometer with a Cu anode equipped with a Ni filter in the Bragg–Brentano geometry. The samples were prepared by drop-casting a suspension of the NPs onto a Si(111) zero background sample holder. Data from each sample were measured from 5–80° 2θ.

Pair distribution function analysis

Total scattering measurements were performed at the beamline 11-ID-B at the Advanced Photon Source, Argonne National Laboratory. The samples were loaded into NMR tubes with a diameter of 3 mm and thickness of 0.27 mm and placed in dedicated holders. The data were collected at a wavelength of λ = 0.2113 Å using a 2D PerkinElmer detector with a pixel size of 200 × 200 μm in the rapid acquisition pair distribution function setup [1]. The detector distances were calibrated from a CeO_2 standard in Fit2D [2] and the 2D images were integrated using Dioptas [3]. Background subtraction and Fourier transform into the pair distribution function (PDF) were done using the xPDFsuite with a Qmax of 21 Å^{-1} for the samples containing precursors and 23.8 Å^{-1} for samples containing Os NPs [4]. The obtained PDFs were analyzed using PDFgui [5] and Diffpy-CMI [6]. The metallic cluster models were generated using the ASE package [7].

The fitting procedure of the Os NPs was performed by generating a large number of different Os clusters, including simple cubic, fcc, bcc, and hcp, as well as icosahedra, octahedra, decahedra, and Wulff constructions with different combinations of surfaces and potentials [7]. Each cluster was fitted to the data by

refining a scale factor, a single atomic displacement parameter for all the atoms and a correlated motion parameter, δ_2. Furthermore, a lattice for each cluster was also refined, which allows the cluster to expand and contract isotropically [8]. Fits using crystalline models were done by refining the scale factor, lattice parameters, a single atomic displacement parameter for all the atoms, and a correlated motion parameter, δ_2. The effects of the NP size were either modelled using a single spherical particle size or a lognormal distribution of spherical particles as implemented in Diffpy-CMI.

Small angle X-ray scattering

Small angle X-ray scattering (SAXS) measurements were performed by depositing a few drops of the formed nanomaterial onto a 5–7 µm thick mica window [9]. After the solvent was dried in a fume cupboard overnight at room temperature, the quartz window was placed in a dedicated sandwich cell for measurements. The measurements were performed at the Niels Bohr Institute at the University of Copenhagen using a SAXSLab instrument (JJ-XRay) with a Rigaku 100 XL + micro focus sealed X-ray producing a photon beam with a wavelength of 1.54 Å tube and a Dectris 2D 300 K Pilatus detector. The two-dimensional scattering data were azimuthally averaged, normalized by the incident radiation intensity, sample exposure time, and transmission using the Saxsgui software. Measurements were performed under vacuum.

The radially averaged intensity $I(q)$ is given as a function of the scattering vector $q = 4\pi\cdot\sin(\theta)/\lambda$, where λ is the wavelength and 2θ is the scattering angle. The background-corrected scattering data were fitted using a power law, taking into account the behavior at low q value and a model of polydispersed spheres described by a volume-weighted

lognormal distribution. Better fits were obtained using a second model of polydispersed spheres also described by a volume-weighted lognormal distribution and large size distribution probably accounting for the challenging background subtraction. A structure factor contribution was needed to properly model the data. We employed a hard-sphere structure factor $F(R,\eta)$ as described in Reference [10]. The scattering data are fitted to the following general expression:

$$I(q) = A\cdot q^{-n} + C_1 \cdot F(R_1, \eta_1) \cdot \int P_{S1}(q, R)\, V_1(R)\, D_1(R)\, dR + C_b \cdot \int P_{Sb}(q, R)\, V_b(R)\, D_b(R)\, dR$$

where $A\cdot q^{-n}$ corresponds to the power law where A and n are free parameters; C_1 and C_b are scaling constants, P_{S1} and P_{Sb} the sphere form factors, V_1 and V_b the particle volumes and D_1 and D_b the lognormal size distribution. The sphere form factor [11] is given by:

$$P_s(q,R)= \left(3\,\frac{\sin(qR)-qR\cos(qR)}{(qR)^3}\right)^2$$

and the lognormal distribution by:

$$D(R)=\frac{1}{R\sigma\sqrt{2\pi}}\exp\left(\frac{-\left[\ln\left(\frac{R}{R_0}\right)\right]^2}{2\sigma^2}\right)$$

where σ is the variance and R_0 the geometric mean of the lognormal distribution. The fitting was done using home-written MATLAB code. The free parameters in the model are: A, n, R_1, R_b, σ_1, σ_b, C_1, C_b, η_1. The values obtained for these parameters are reported in Table S2.

The average volume of NP from population 1 and from population b, $<V>_1$ and $<V>_b$ respectively, lead to the definition of the volume fraction of population 1, Φ_{V1}, and the volume fraction of population b, Φ_{Vb}, as:

$$\Phi_{V1} = \frac{N_1 <V>_1}{N_1 <V>_1 + N_b <V>_b} = 1 - \Phi_{Vb}$$

$$\frac{\Phi_{V1}}{\Phi_{Vb}} = \frac{N_1 < V >_1}{N_b < V >_b}$$

$$\frac{N_1}{N_b} = \frac{\Phi_{V1} \ < V >_b}{\Phi_{Vb} \ < V >_1}$$

where N_1 and N_b are the number of NPs in the population 1 or b, respectively.

From the SAXS data acquisition we have the relationship between the retrieved coefficient C_1 and C_b given by $C_i = k \cdot \Phi_{Vi}. <V>_i$ where I = 1 or b and k is a constant.

$$k = \frac{C_1}{\Phi_{V1} < V >_1} = \frac{C_b}{\Phi_{Vb} < V >_b} = \frac{C_b}{(1 - \Phi_{V1}) < V >_b}$$

$$\frac{\Phi_{V1}}{1 - \Phi_{V1}} = \frac{C_1 < V >_b}{C_b < V >_1}$$

$$\Phi_{V1} = \frac{1}{1 + \ \frac{C_b < V >_1}{C_1 < V >_b}}$$

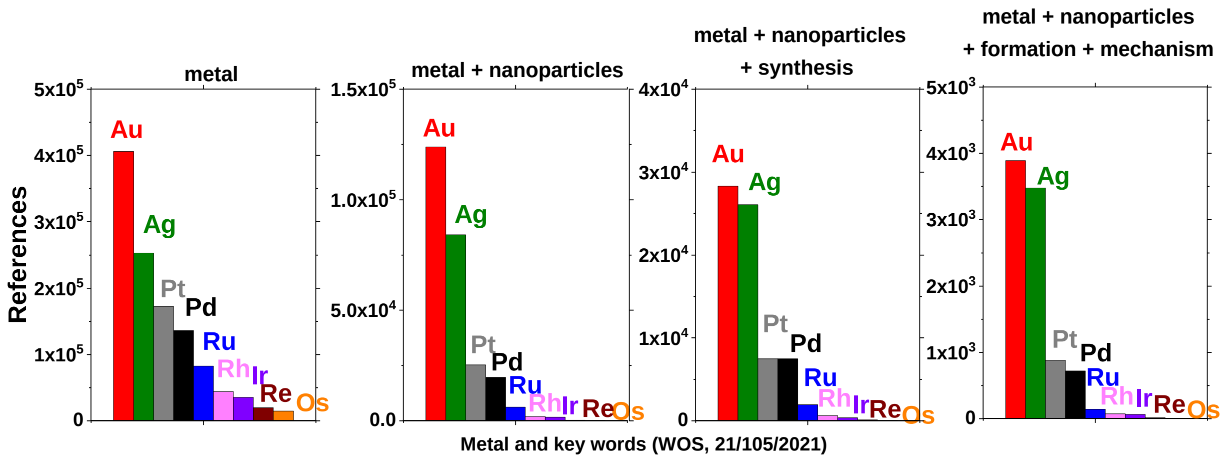

Key words	Au	Ag	Pt	Pd	Ru	Rh	Ir	Re	Os
A: metal	406181	252832	172413	136236	82525	43919	35286	19507	14559
A + Bs: nanoparticles	123885	84094	25313	19727	6175	1870	1708	333	186
A + B	36879	20908	5168	3592	1051	400	297	60	37
A + C:synthesis	42121	40049	23036	47554	22294	12909	7099	4023	2642
A + Bs + C	28290	26083	7467	7463	1956	615	367	95	45
A + B + C	6796	5465	1445	1346	317	127	50	14	8
A + Bs + D:formation	18289	15325	3962	3748	951	386	288	70	36
A + B + D	5371	3802	880	766	200	87	62	7	3
A + Bs + E:mechanism	16239	13484	3550	2371	715	235	263	58	24
A + B + E	4714	3274	760	463	153	49	54	8	1
A + Bs + D + E	3892	3477	882	720	142	75	65	14	5

Figure S1: Number of references retrieved from the Web of Science (WOS) database on 21/05/2021 for different metals (Au: Gold, Ag: Silver, Pt: Platinum, Pd: Palladium, Ru: Ruthenium, Rh: Rhodium, Ir: Iridium, Re: Rhenium, Os: Osmium) and different keywords and combination of keywords, as indicated. A = metal; B = nanoparticle; C = synthesis; D = formation; E = mechanism.

Table S1: Os NP synthesis parameters and resulting morphology and size for 2.5 mM Os and NaOH/Os of 20 or 0 obtained by leaving the reaction mixture at 90 °C in closed polypropylene containers for 6 h

Sample	Precursor	Solvent	v.% H_2O	With NaOH			Without base		
				Colour	Form	Figure	Colour	Form	Figure
M1	$OsCl_3$	M	-	Blue	NPs	S2	Brown	Network	S6
M1-10	$OsCl_3$	M	10	Blue Black$_{(s)}$	Network	S2	-	-	-
M1-25	$OsCl_3$	M	25	Blue Black$_{(s)}$	Network	S2	Blue Black$_{(s)}$	NPs	S6
M1-50	$OsCl_3$	M	50	Black$_{(s)}$	Network	S2	Blue Black$_{(s)}$	NPs	S6
M1-75	$OsCl_3$	M	75	Black$_{(s)}$	Network	S2	Blue Black$_{(s)}$	Network	S6
M2	H_2OsCl_6	M	-	Blue	NPs	S3	Brown	Network	S7
M2-10	H_2OsCl_6	M	10	Blue Black$_{(s)}$	Network	S3	-	-	-
M2-25	H_2OsCl_6	M	25	Blue Black$_{(s)}$	Network	S3	Blue Black$_{(s)}$	NPs	S7
M2-50	H_2OsCl_6	M	50	Black$_{(s)}$	Network	S3	Blue Black$_{(s)}$	NPs	S7
M2-75	H_2OsCl_6	M	75	Black$_{(s)}$	Network	S3	Blue Black$_{(s)}$	NPs	S7
E1	$OsCl_3$	E	-	Black$_{(s)}$	NPs-Network	S4	Dark	NPs*	S8
E1-10	$OsCl_3$	E	10	Black$_{(s)}$	NPs-Network	S4	-	-	-
E1-25	$OsCl_3$	E	25	Blue Black$_{(s)}$	Network	S4	Blue Black$_{(s)}$	NPs	S8
E1-50	$OsCl_3$	E	50	Black$_{(s)}$	Network	S4	Blue Black$_{(s)}$	NPs	S8
E1-75	$OsCl_3$	E	75	Black$_{(s)}$	Network	S4	Blue Black$_{(s)}$	Network	S8
E2	H_2OsCl_6	E	-	Black$_{(s)}$	NPs-Network	S5	Dark	-	S9
E2-10	H_2OsCl_6	E	10	Black$_{(s)}$	Network	S5	-	-	-
E2-25	H_2OsCl_6	E	25	Black$_{(s)}$	Network	S5	Blue Black$_{(s)}$	NPs	S9
E2-50	H_2OsCl_6	E	50	Black$_{(s)}$	Network	S5	Blue Black$_{(s)}$	NPs	S9
E2-75	H_2OsCl_6	E	75	Black$_{(s)}$	Network	S5	Blue Black$_{(s)}$	NPs	S9
W1-100	$OsCl_3$	M	100	Transparent	-	-	Black$_{(s)}$	-	S6, S8
W2-100	H_2OsCl_6	M	100	Transparent	-	-	Black$_{(s)}$	NPs	S7, S9

M: methanol; E: ethanol; "Black$_{(s)}$" means that a darker solid structure can be seen after reaction; * means that the identified structure was challenging to find on the grid; "Network" refers to structures that are not individuals NPs or to NPs that seem to be embedded in a polymer-like film.

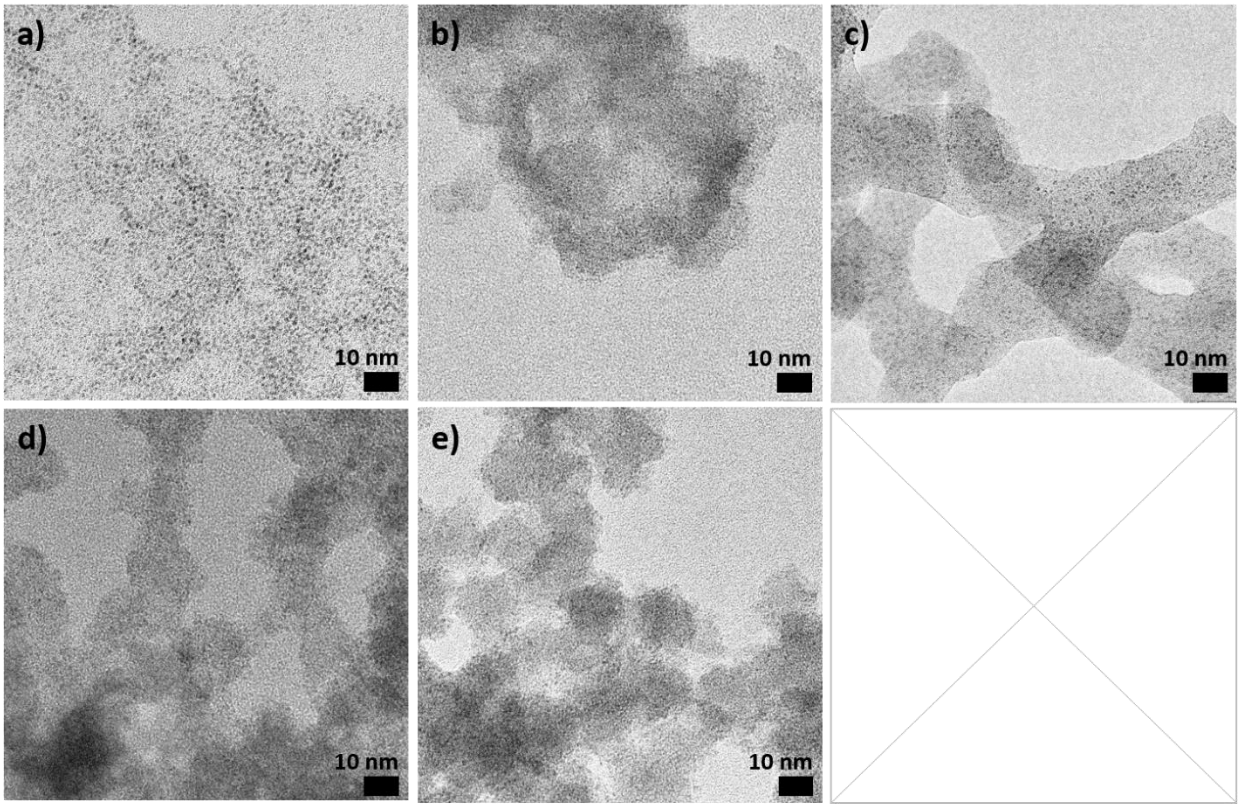

Figure S2: TEM micrographs of Os nanomaterials obtained in alkaline methanol using $OsCl_3$ as the precursor with (a) 0 vol %, (b) 10 vol % (c) 25 vol %, (d) 50 vol %, (e) 75 vol % water. Nothing could be observed on the grid for 100 vol % water.

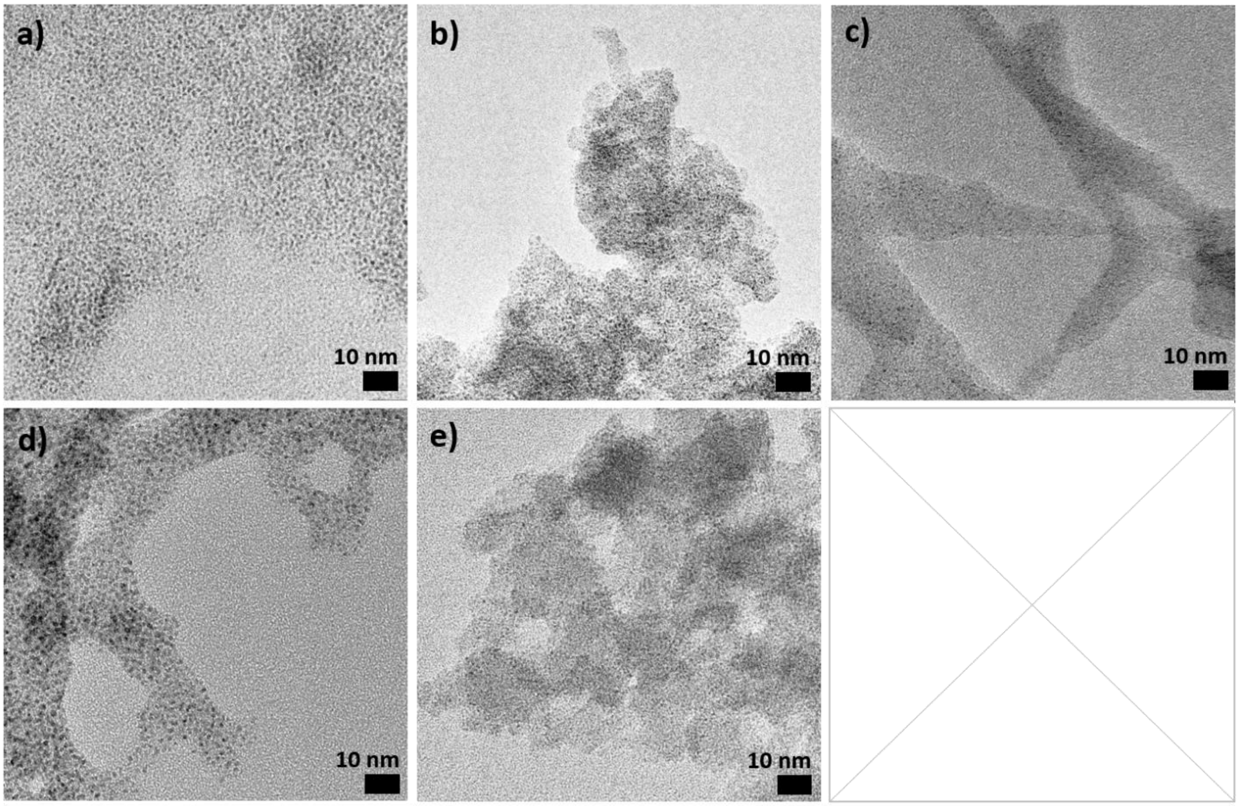

Figure S3: TEM micrographs of Os nanomaterials obtained in alkaline methanol using H_2OsCl_6 as the precursor with (a) 0 vol %, (b) 10 vol % (c) 25 vol %, (d) 50 vol %, (e) 75 vol % water. Nothing could be observed on the grid for 100 vol % water.

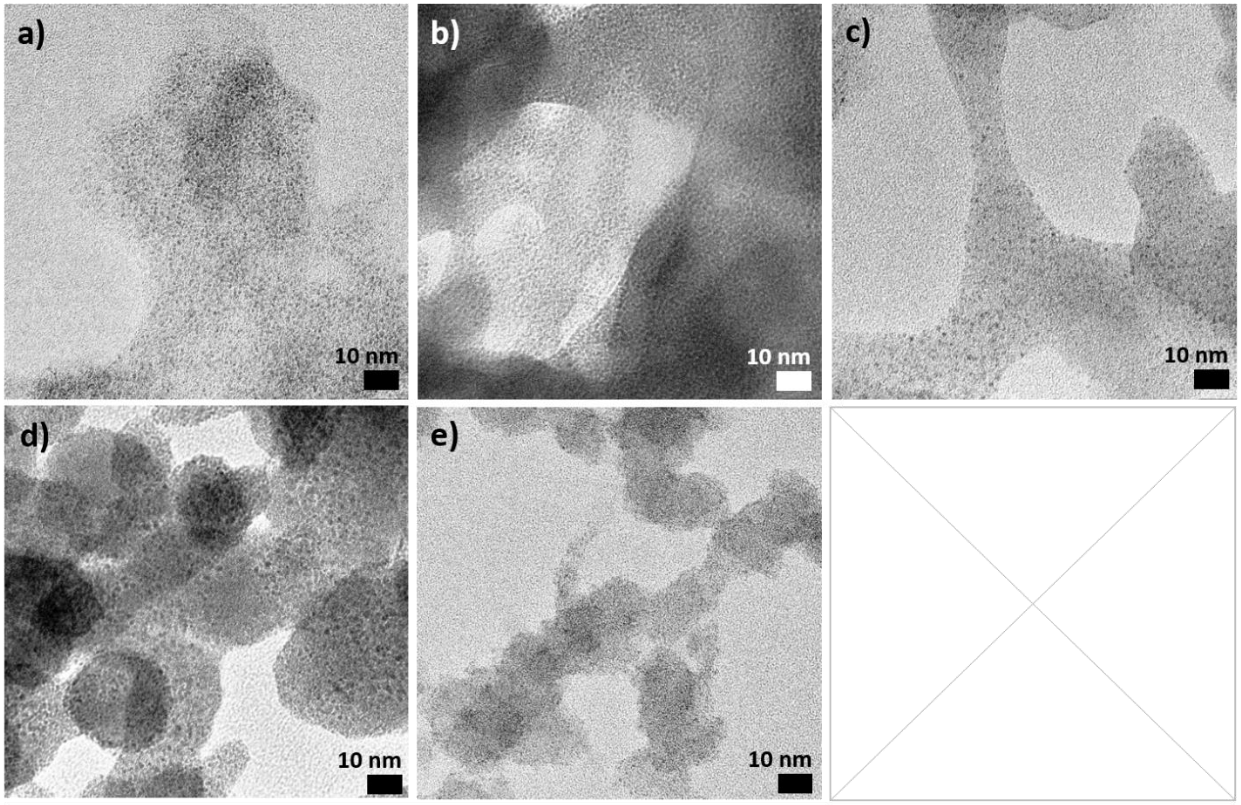

Figure S4: TEM micrographs of Os nanomaterials obtained in alkaline ethanol using $OsCl_3$ as the precursor with (a) 0 vol %, (b) 10 vol % (c) 25 vol %, (d) 50 vol %, (e) 75 vol % water. Nothing could be observed on the grid for 100 vol % water.

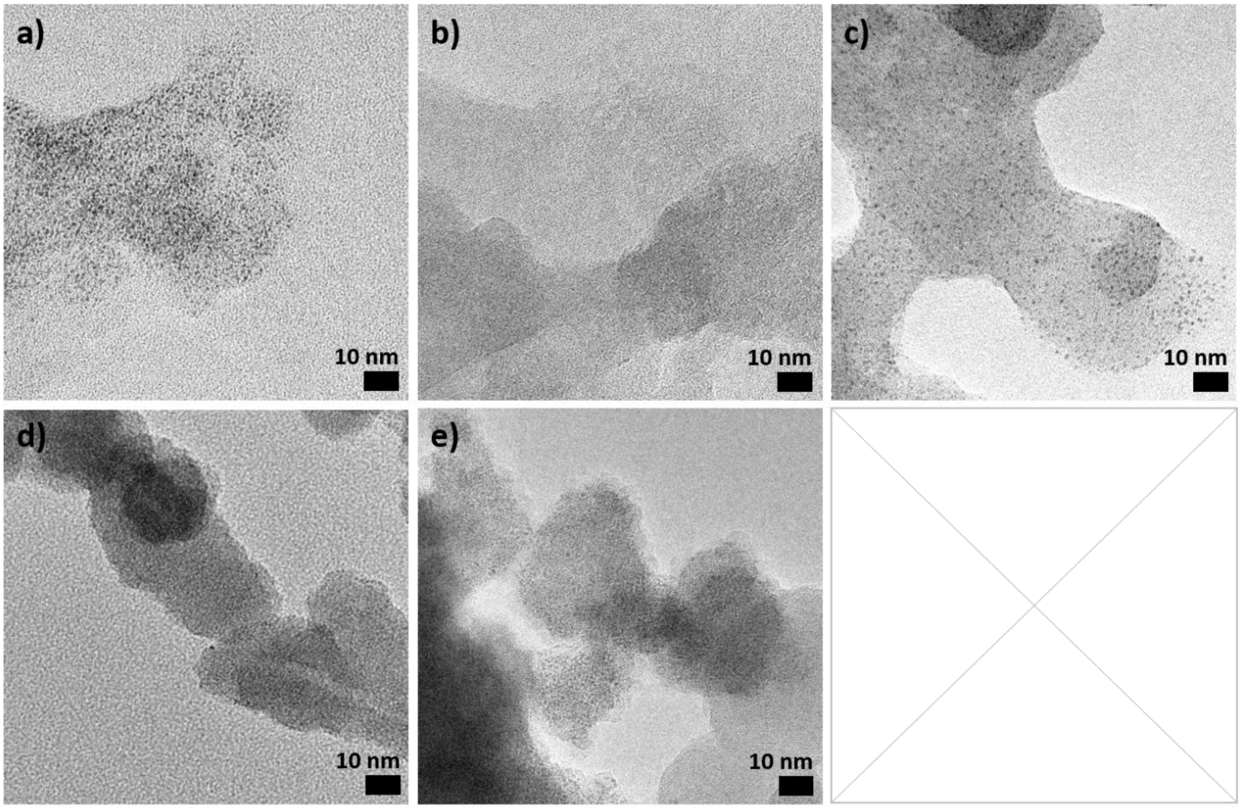

Figure S5: TEM micrographs of Os nanomaterials obtained in alkaline ethanol using H_2OsCl_6 as the precursor with (a) 0 vol %, (b) 10 vol % (c) 25 vol %, (d) 50 vol %, (e) 75 vol % water. Nothing could be observed on the grid for 100 vol % water.

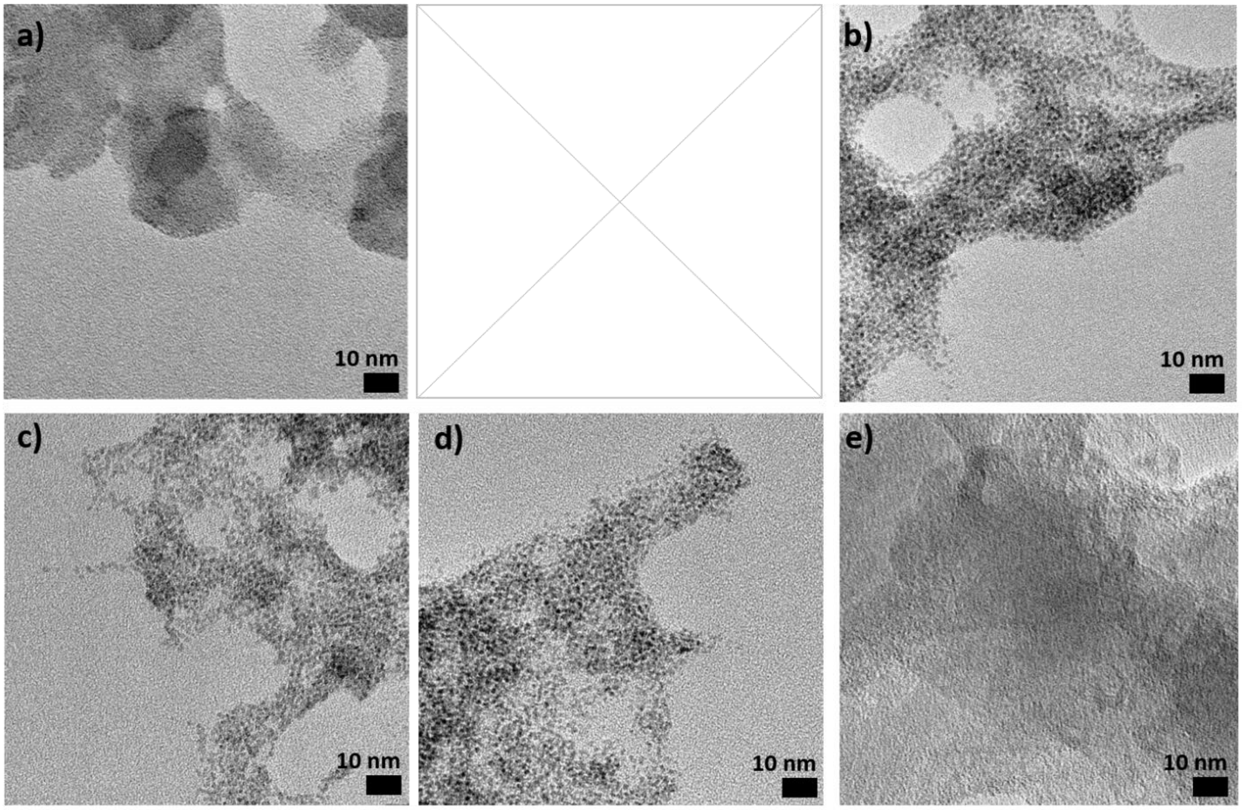

Figure S6: TEM micrographs of Os nanomaterials obtained in methanol (no base) using $OsCl_3$ as the precursor with (a) 0 vol %, (b) 25 vol % (c) 50 vol %, (d) 75 vol %, (e) 100 vol % water.

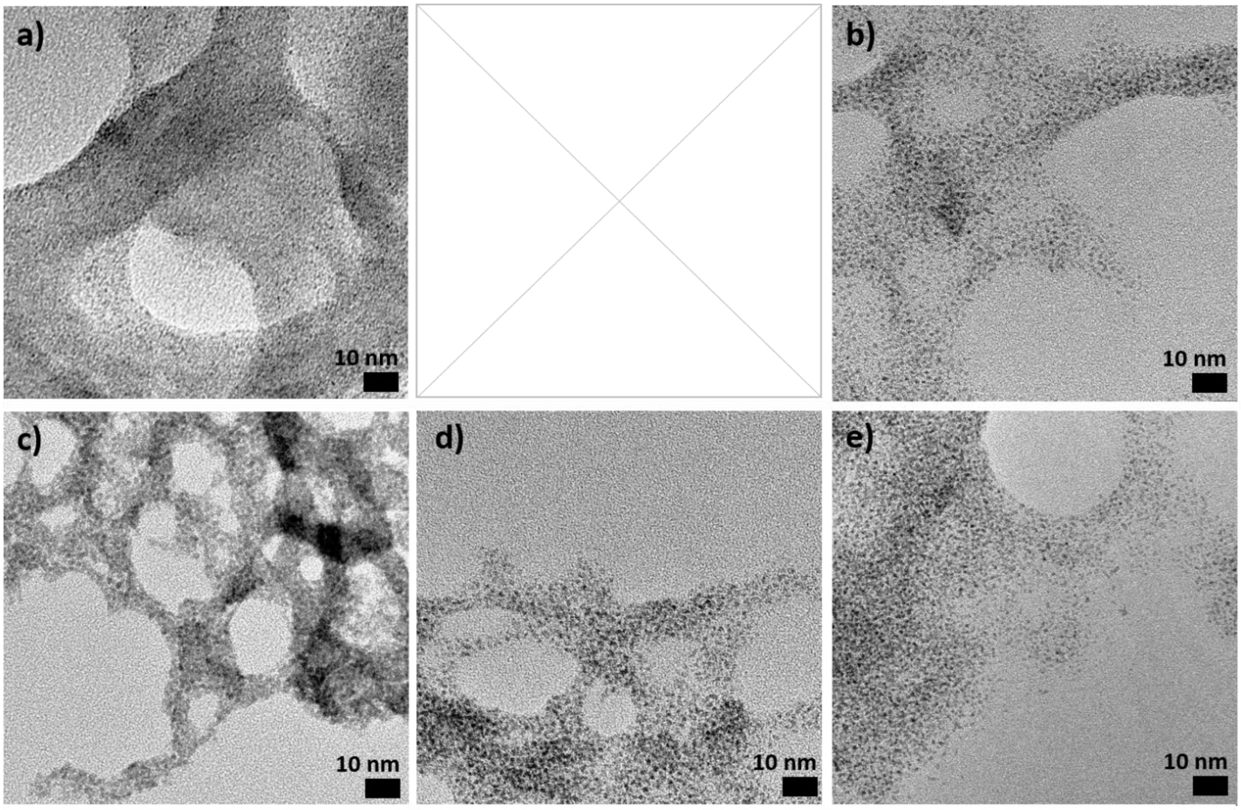

Figure S7: TEM micrographs of Os nanomaterials obtained in methanol (no base) using H_2OsCl_6 as the precursor with (a) 0 vol %, (b) 25 vol % (c) 50 vol %, (d) 75 vol %, (e) 100 vol % water.

Some NPs could be observed even with 100 vol % water but the spots and the grids were scarce which might indicate that the NPs formed from H_2OsCl_6 upon drying on the TEM grid. The lack of formation of NPs in the absence of alcohols playing the role of reducing agent is expected.

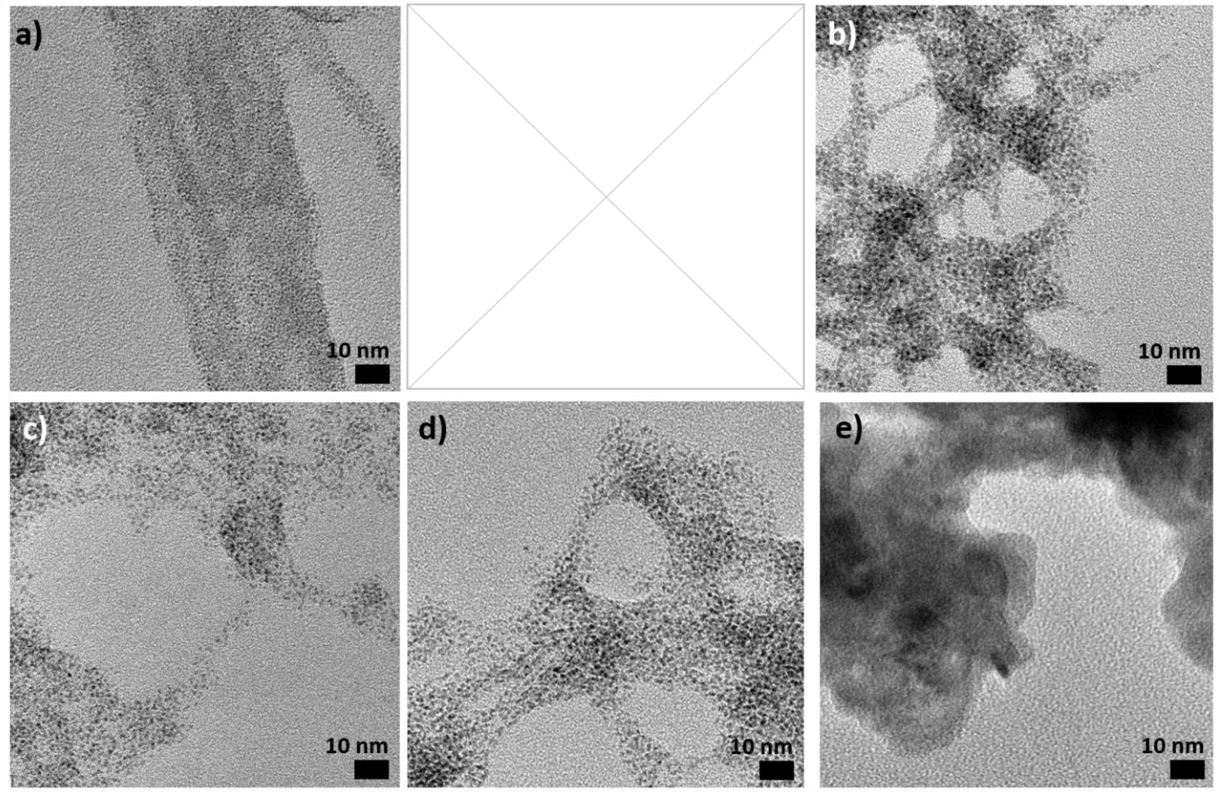

Figure S8: TEM micrographs of Os nanomaterials obtained in ethanol (no base) using $OsCl_3$ as the precursor with (a) 0 vol %, (b) 25 vol % (c) 50 vol %, (d) 75 vol %, (e) 100 vol % water.

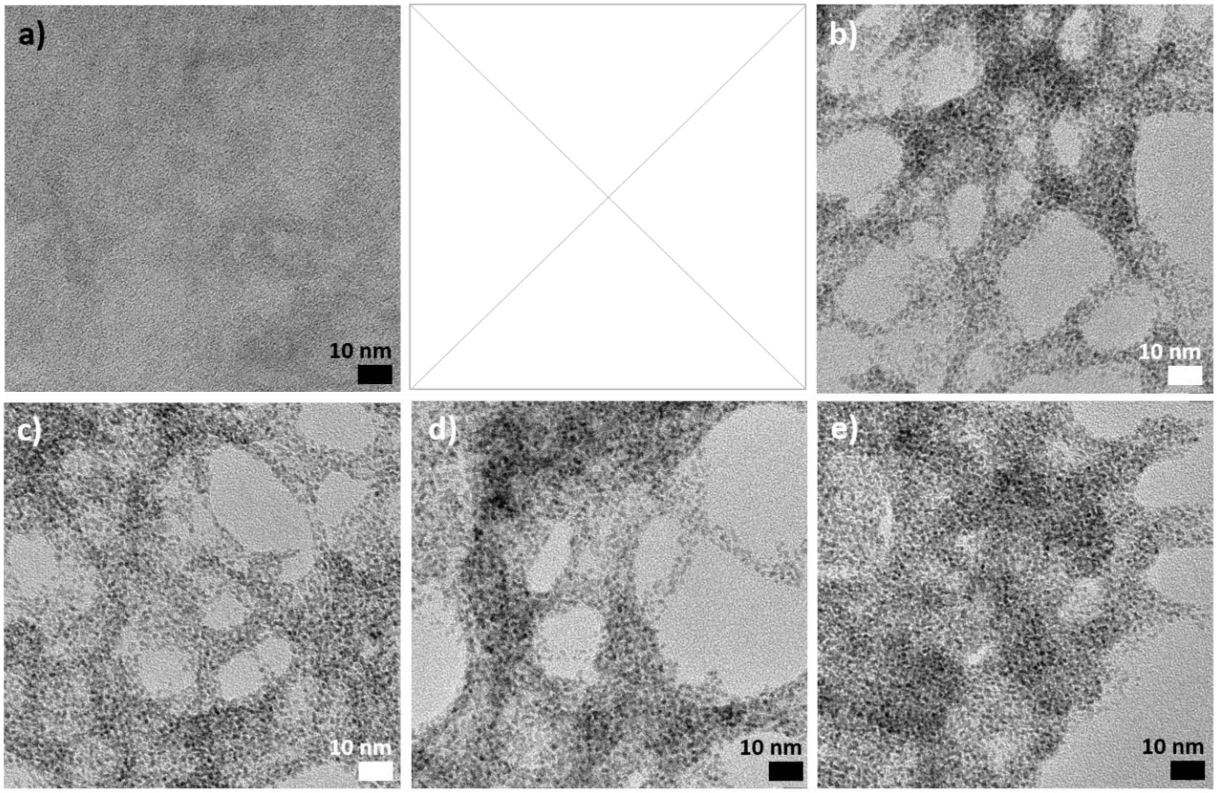

Figure S9: TEM micrographs of Os nanomaterials obtained in ethanol (no base) using H_2OsCl_6 as the precursor with (a) 0 vol %, (b) 25 vol % (c) 50 vol %, (d) 75 vol %, (e) 100 vol % water.

Some NPs could be observed even with 100 vol % water but the spots and the grids were scarce which might have indicated that the NPs formed from H_2OsCl_6 upon drying on the TEM grid. The lack of formation of NPs in absence of alcohols playing the role of reducing agent is expected.

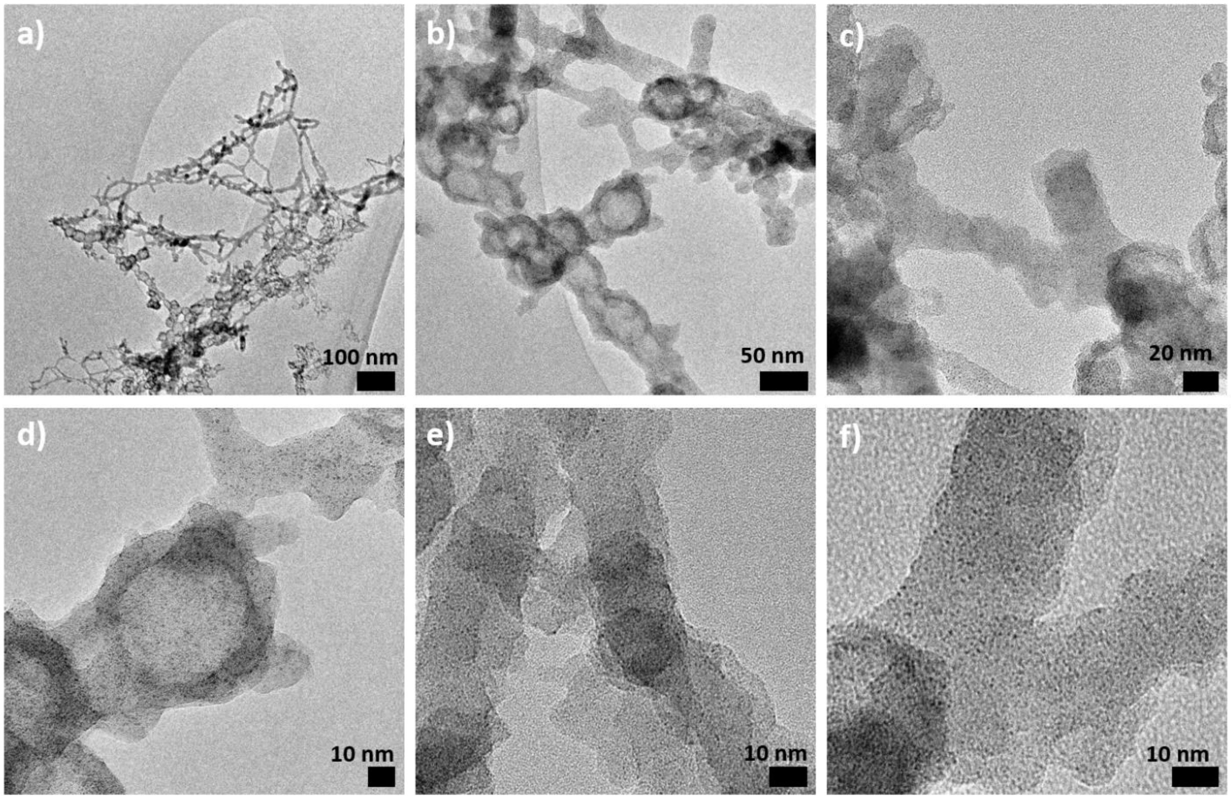

Figure S10: TEM micrographs of Os nanomaterials obtained in alkaline methanol using $OsCl_3$ as the precursor with 25 vol % of water at different magnifications as indicated.

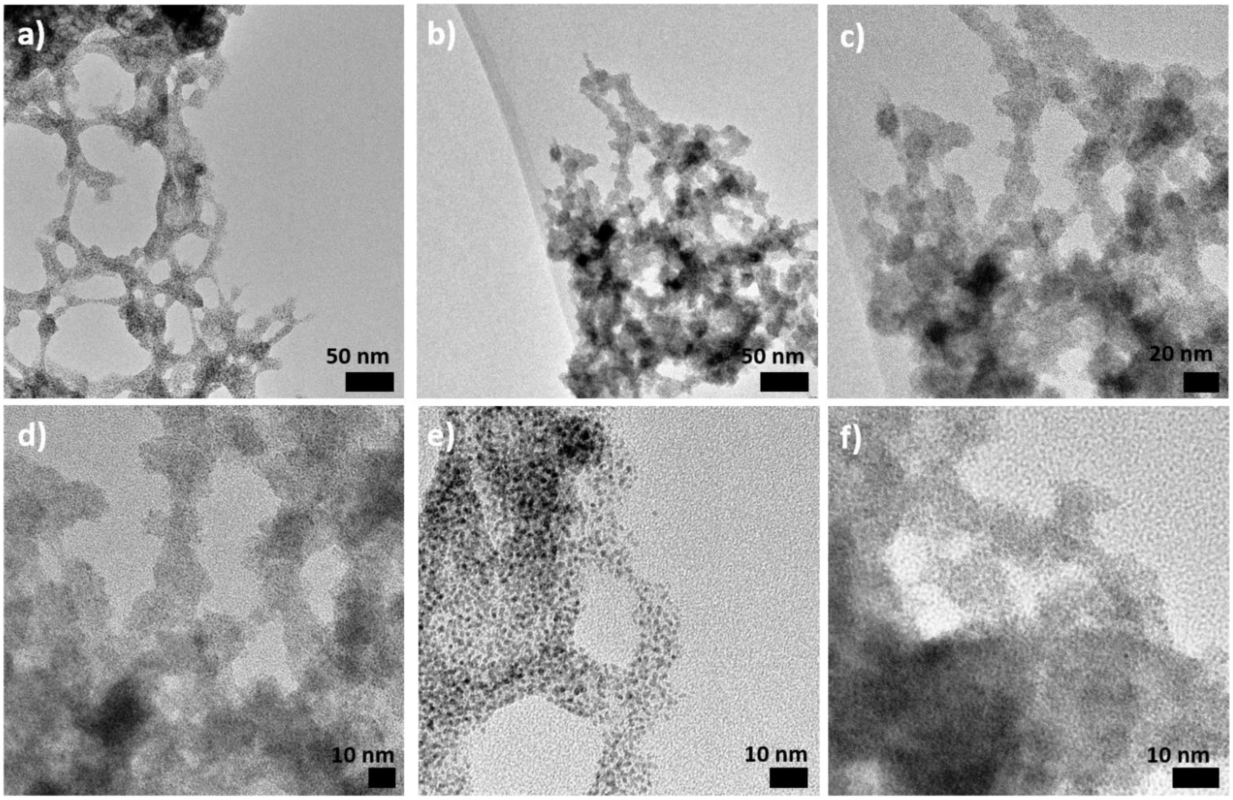

Figure S11: TEM micrographs of Os nanomaterials obtained in alkaline methanol using $OsCl_3$ as the precursor with 50 vol % of water at different magnifications as indicated.

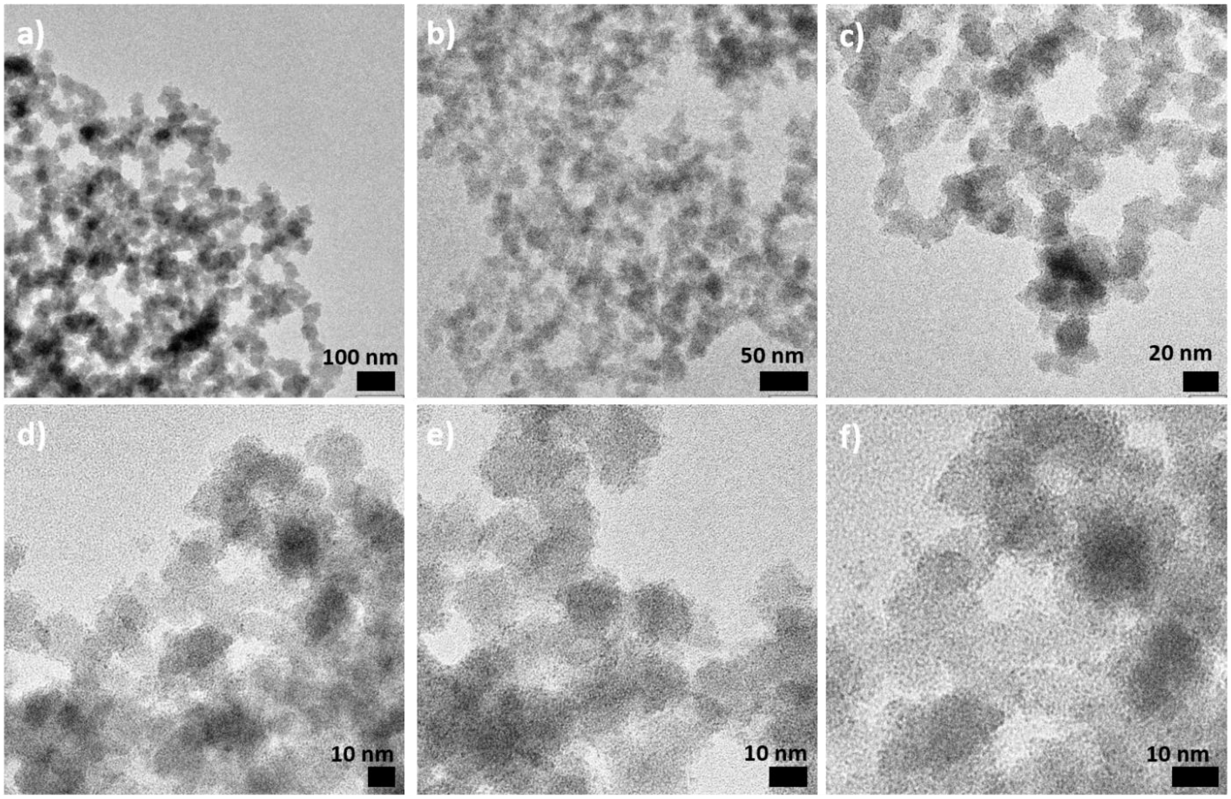

Figure S12: TEM micrographs of Os nanomaterials obtained in alkaline methanol using $OsCl_3$ as the precursor with 75 vol % of water at different magnifications as indicated.

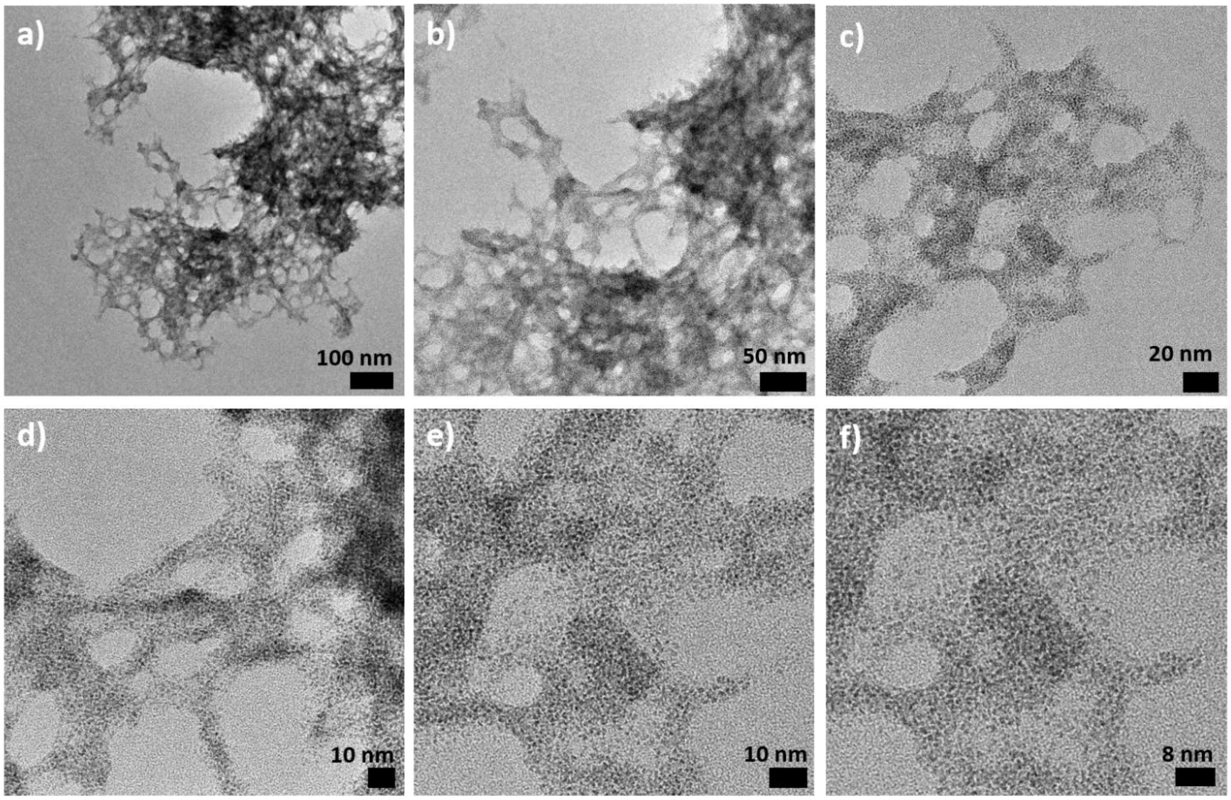

Figure S13: TEM micrographs of Os nanomaterials obtained in methanol (no base) using $OsCl_3$ as the precursor with 25 vol % of water at different magnifications as indicated.

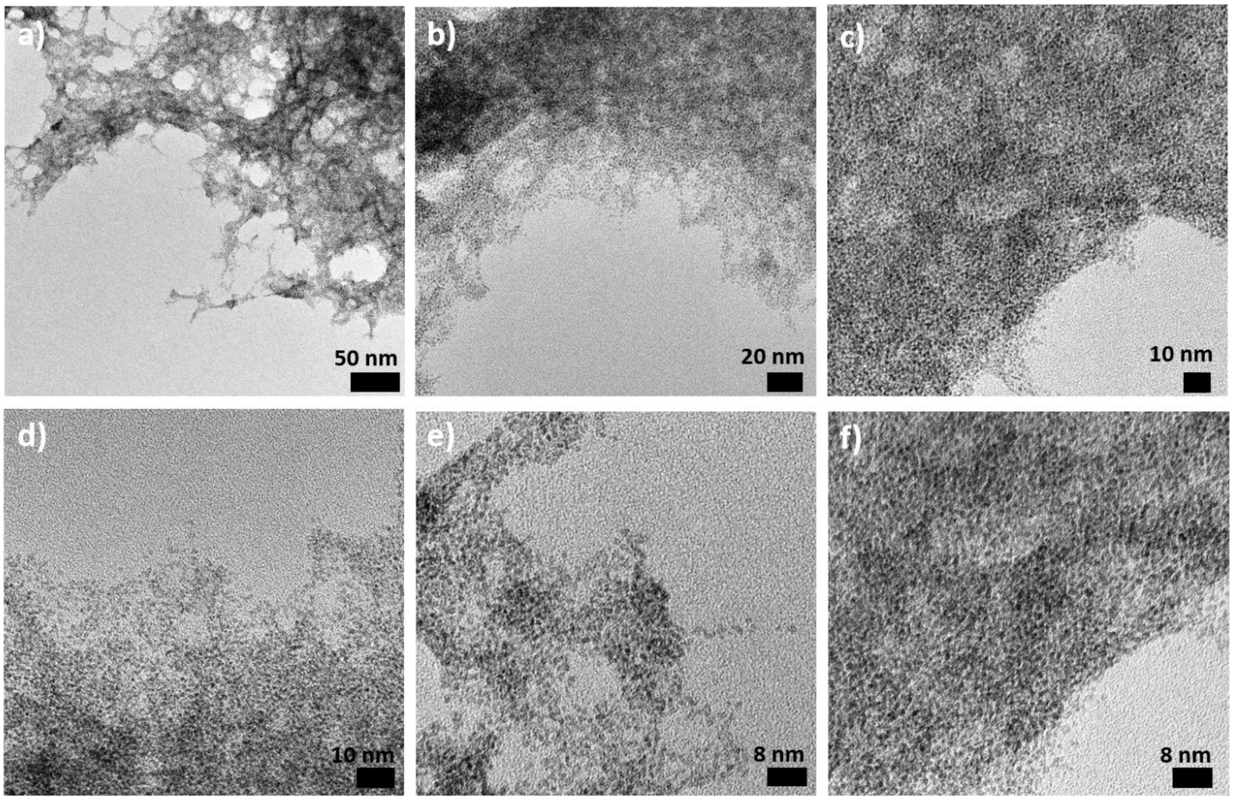

Figure S14: TEM micrographs of Os nanomaterials obtained in methanol (no base) using $OsCl_3$ as the precursor with 50 vol % of water at different magnifications as indicated.

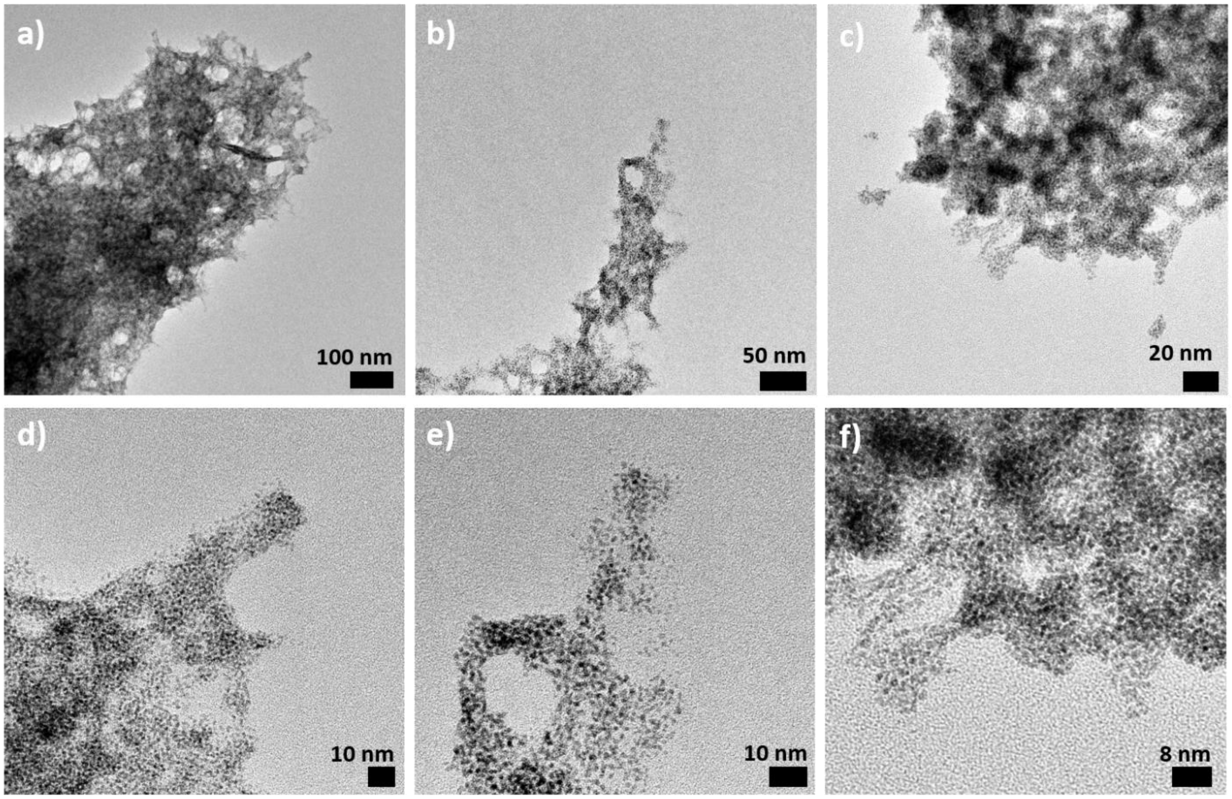

Figure S15: TEM micrographs of Os nanomaterials obtained in methanol (no base) using $OsCl_3$ as the precursor with 75 vol % of water at different magnifications as indicated.

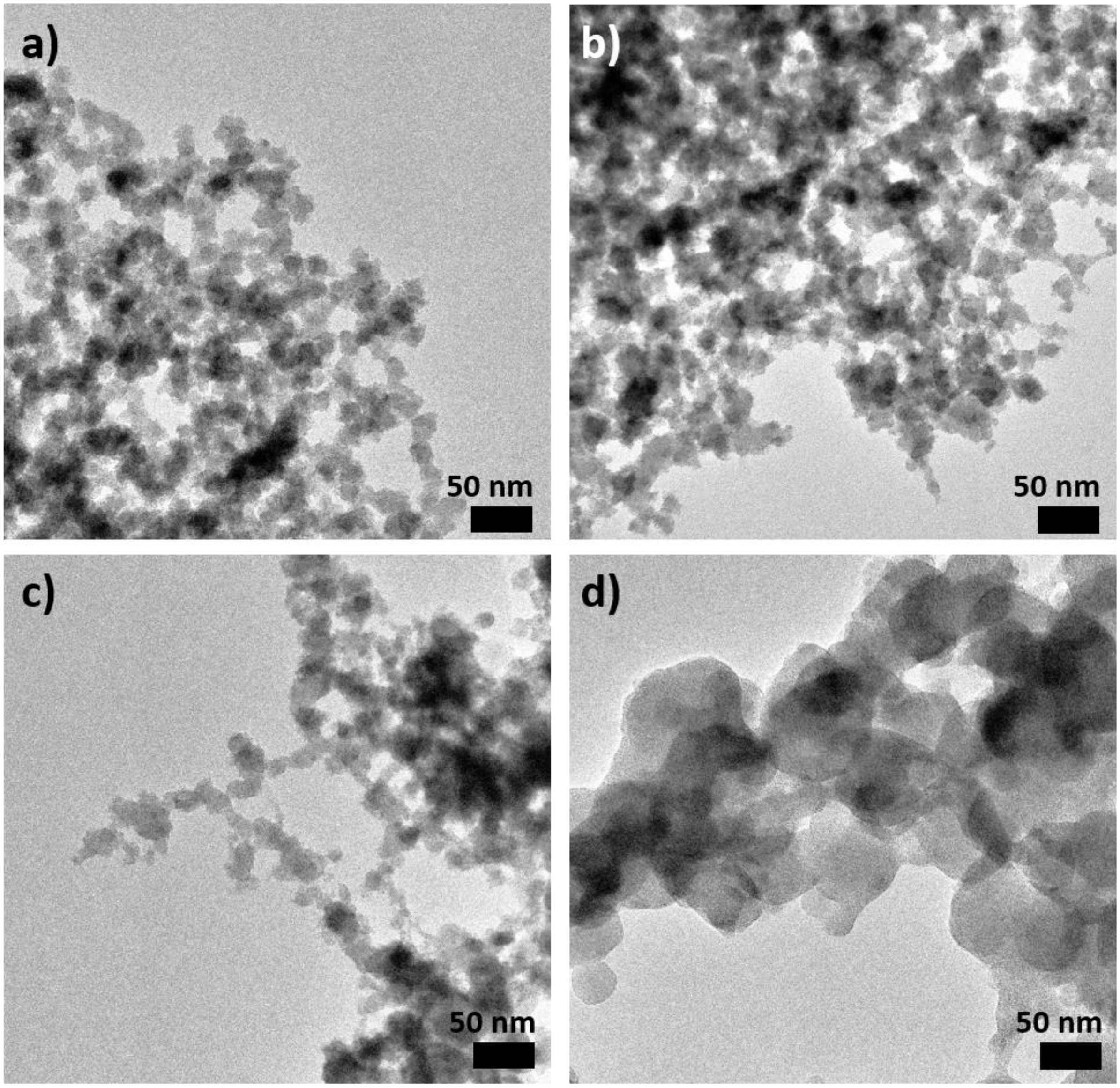

Figure S16: TEM micrographs of Os nanomaterials obtained in alkaline solvent and 75 vol % water using (a) $OsCl_3$ in alkaline methanol, (b) H_2OsCl_6 in alkaline methanol, (c) $OsCl_3$ in alkaline ethanol, and (d) H_2OsCl_6 in alkaline ethanol.

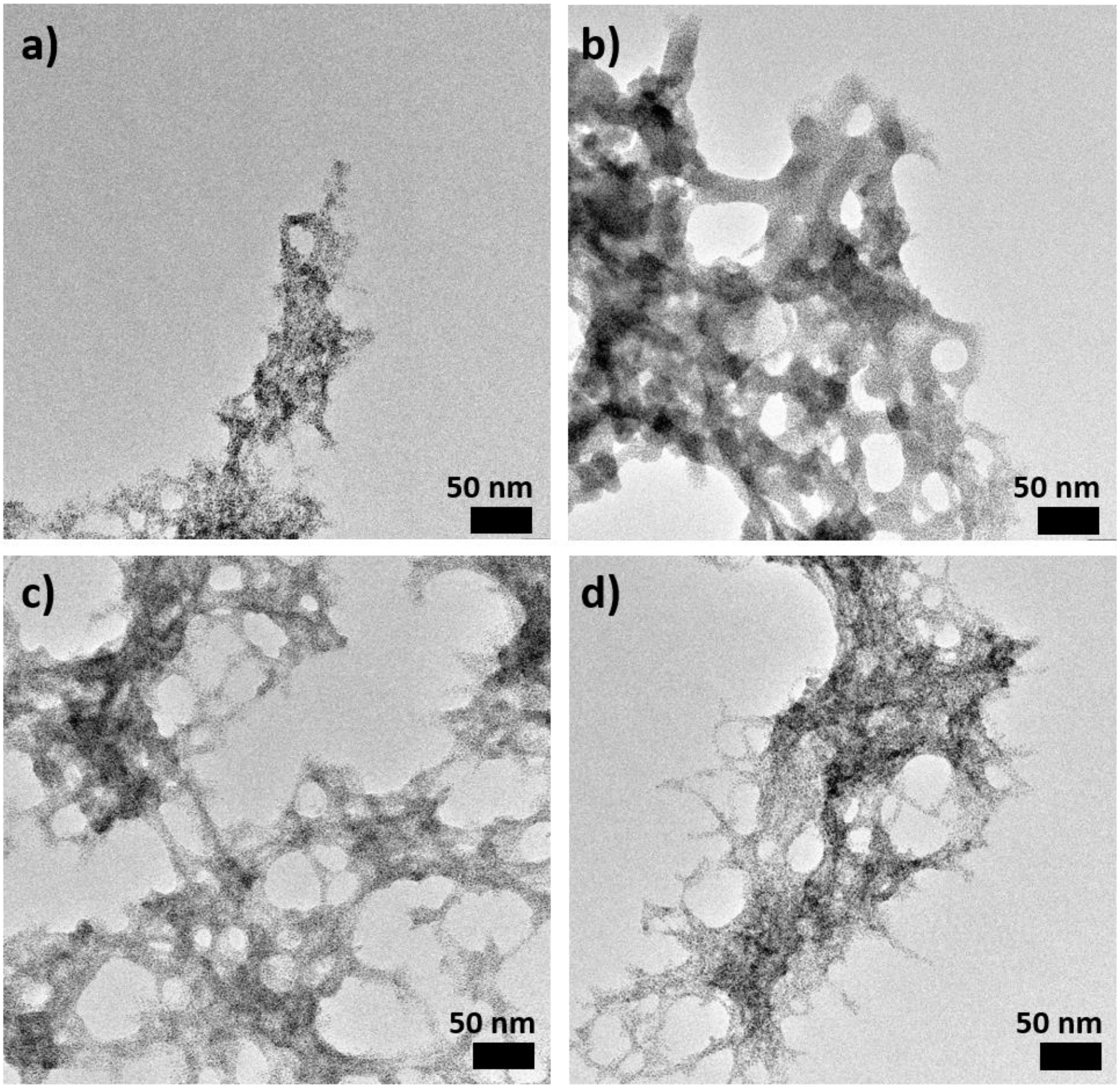

Figure S17: TEM micrographs of Os nanomaterials obtained in solvent without base and 75 vol % water using (a) $OsCl_3$ in alkaline methanol, (b) H_2OsCl_6 in alkaline methanol, (c) $OsCl_3$ in alkaline ethanol and (d) H_2OsCl_6 in alkaline ethanol.

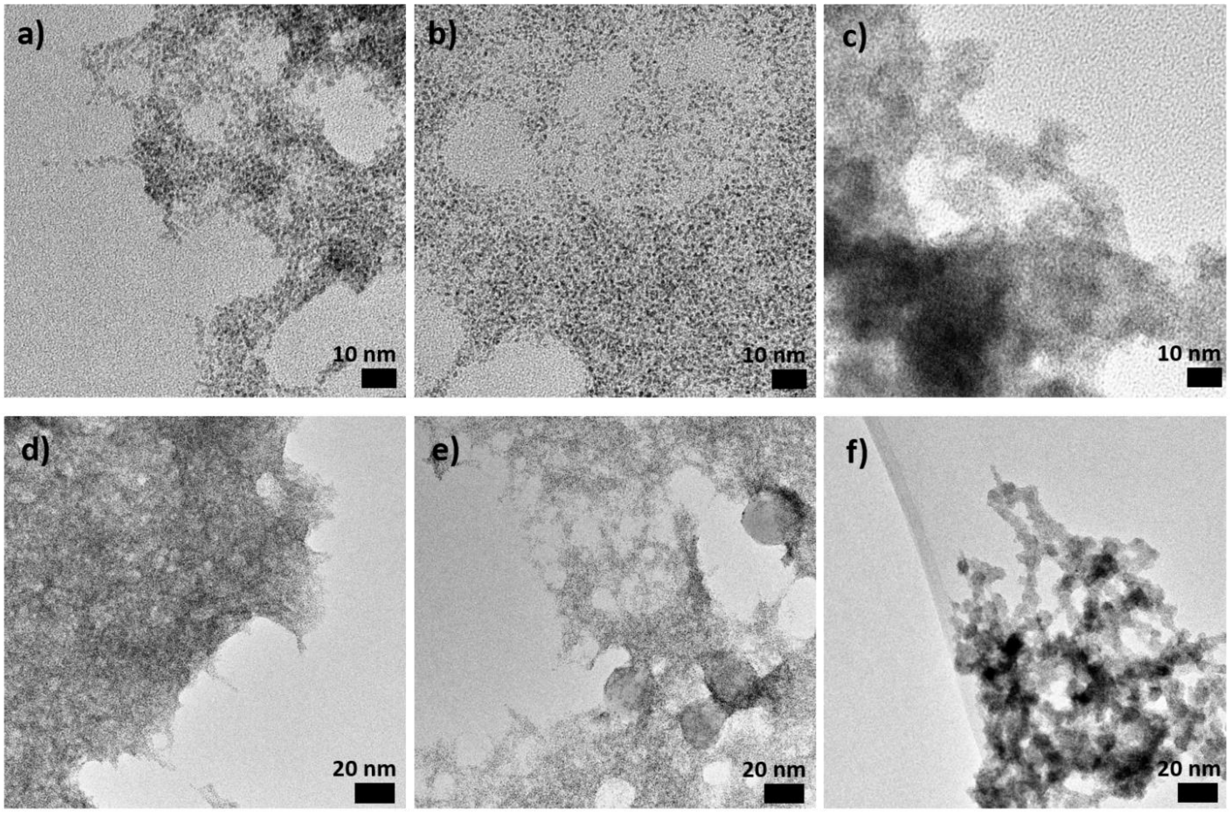

Figure S18: (a–f) TEM micrographs of Os nanomaterials obtained in methanol and 50 vol % water using $OsCl_3$ with NaOH/Os at molar ratios of (a,d) 0, (b,e) 5 and (c,f) 20.

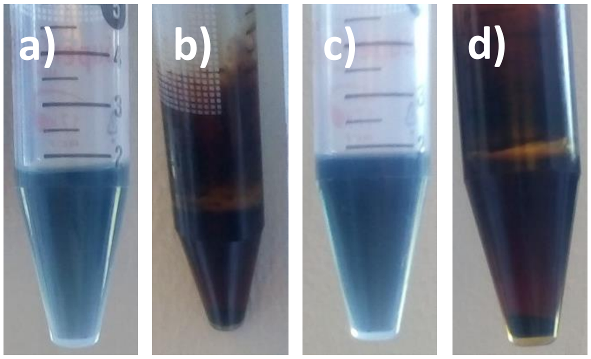

Figure S19: Pictures of alkaline Os solutions after 6 h at 90 °C for (a,b) $OsCl_3$ in (a) alkaline methanol and (b) alkaline ethanol and (c,d) for H_2OsCl_6 in (c) alkaline methanol and (d) alkaline ethanol. The blue colour might indicate the formation of Os complexes and ultimately that the formation of NPs was better achieved using a mixture of alcohol and water as detailed below.

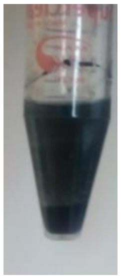

Figure S20: Example of Os solutions after 6 h at 90 °C when no base is used. In this specific case a solution was obtained with $OsCl_3$ and 50:50 (v/v) methanol/water mixture.

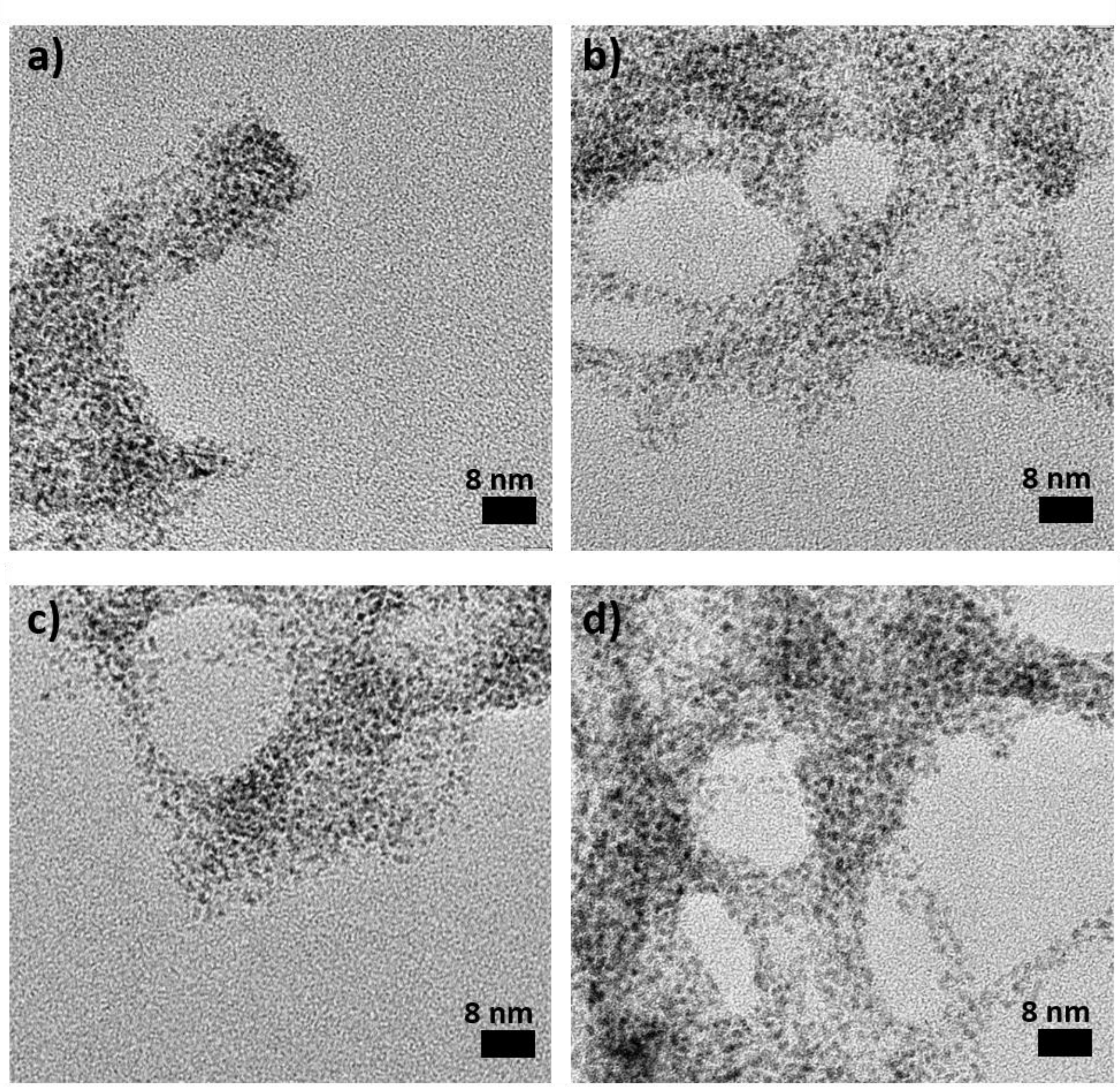

Figure S21: (a–d) TEM micrographs of Os NPs obtained using 75 vol % water in (a,b) methanol, (c,d) ethanol (no base), (a,c) $OsCl_3$ and (b,d) H_2OsCl_6 as precursors.

SAXS

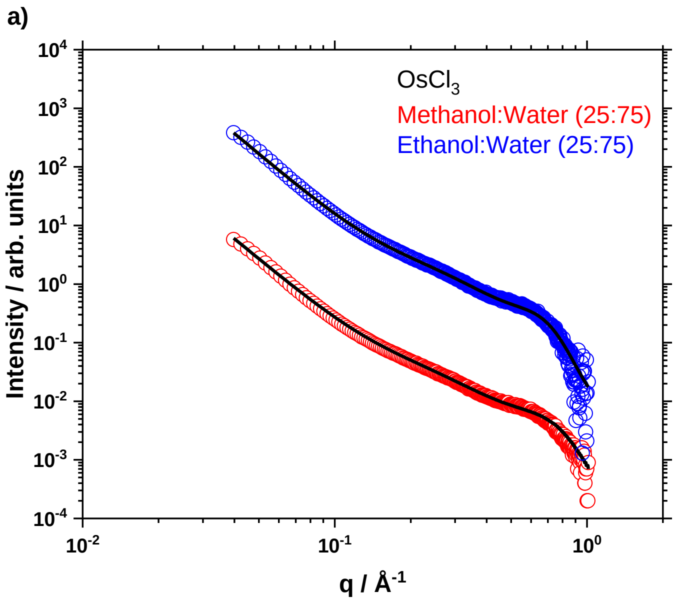

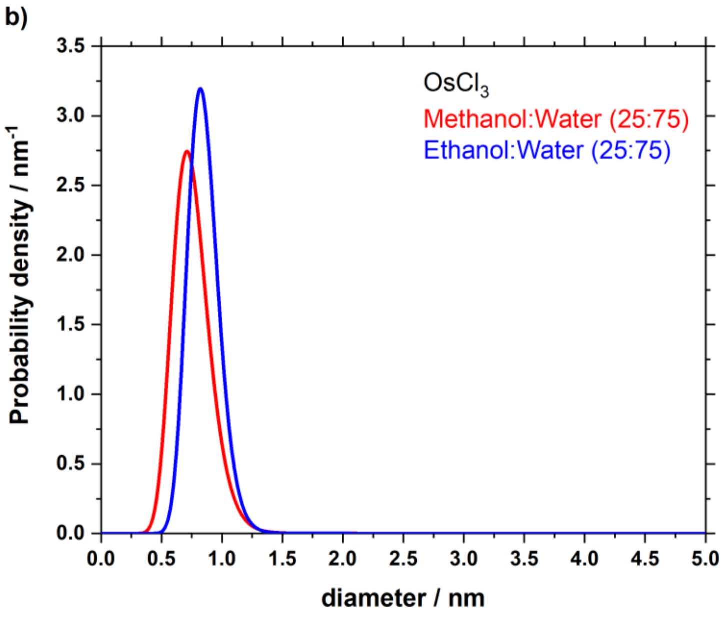

Figure S22: (a) Fits related to the data presented in (b). (b) Probability densities retrieved from the SAXS analysis of the Os NP size. The samples displayed were obtained with 2.5 mM $OsCl_3$ in the absence of a base for a methanol/water mixture of 1:3 in volume after 6 h at 90 °C for a total volume of 2 mL.

Table S2: Fit parameters corresponding to the data displayed in Figure S22.

Sample	Power law		1				b			Size and distribution	
	A x 10^6	n	R_1 (Å)	σ_1	C_1	η_1	R_b (Å)	σ_b	C_b	d_{sample}[A] / nm	σ_{sample}[B] / nm
$OsCl_3$ Methanol:Water (25:75)	60	3.57	3.7	0.2	0.0037	0.17	7	0.30	0.0017	0.8	0.1
$OsCl_3$ Ethanol:Water (25:75)	32	3.62	4.2	0.15	0.0017	0.20	7	0.25	0.0010	0.9	0.1

(A) evaluated as $d_{sample} = 0.2\,\varphi_{V1}.\,e^{\left(\ln(R_1)+\frac{\sigma_1{}^2}{2}\right)} + 0.2\,\varphi_{Vb}.\,e^{\left(\ln(b)+\frac{\sigma_b{}^2}{2}\right)}$

(B) evaluated as $\sigma_{sample} = 0.2\sqrt{\varphi_{V1}{}^2.\left[\left(e^{\sigma_1{}^2}-1\right)e^{\left(2\ln(R_1)+\sigma_1{}^2\right)}\right] + \varphi_{Vb}{}^2.\left[\left(e^{\sigma_b{}^2}-1\right)e^{\left(2\ln(R_b)+\sigma_b{}^2\right)}\right]}$

Samples for PDF analysis

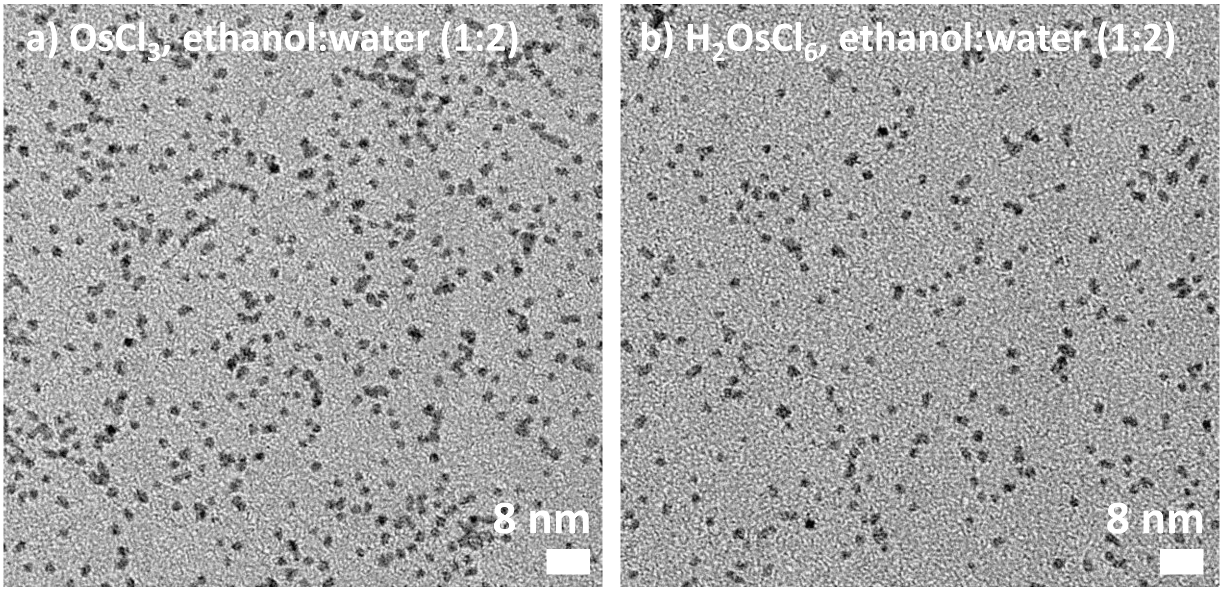

Figure S23: (a,b) TEM micrographs of Os NPs obtained using 66 vol % water and 33 vol % ethanol (no base) and 100 mM of (a) $OsCl_3$ and (b) H_2OsCl_6 as precursors after a one week reaction at 85 °C in NMR tubes (volume approx. 0.2 mL). The size analysis suggest that the NPs are (a) 1.5 ± 0.3 nm and (b) 1.7 ± 0.4 nm.

In line with the small size of the NPs obtained here (in the range of 1–2 nm, see for example Figure 1 and Figure S23), most reported colloidal synthesis methods of Os NPs yield nanomaterials in the size range 1–2 nm. This can further explain why the interest for Os NPs has been limited to date: the small size limits the material characterization that can be done with conventionally available methods. Relatively high-resolution transmission electron microscopy (TEM) is needed to characterize small nanomaterials [12-15] and X-ray diffraction (XRD) analysis is challenging due to the broadening of Bragg

peaks for NPs of approx. 1–2 nm. Due to the very small NP size, XRD analysis of the Os samples actually shows no identifiable Bragg peaks, Figure S24.

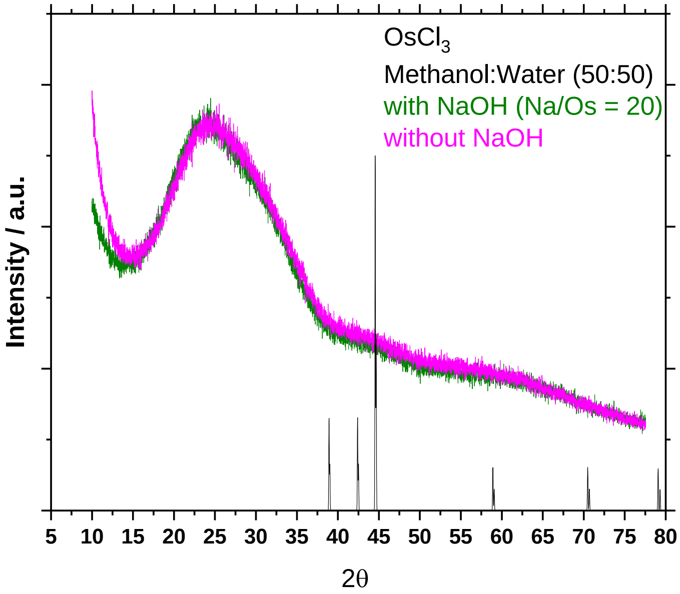

Figure S24: XRD diffractograms for samples prepared from 2.5 mM $OsCl_3$ in a methanol/water 50:50 mixture (in volume) in the presence or absence of a base, as indicated, after 6 h at 90 °C for a total volume of 2 mL. The expected peaks for hcp Os are indicated. The large feature around 25° (2θ) is due to the background support.

The cluster that best fits the PDF obtained, see Figure 2, for NPs formed in methanol/water from H_2OsCl_6 contains 96 Os atoms. The clusters formed from $OsCl_3$ appear slightly smaller, with the best fitting structure for the particles formed in methanol/water containing 72 atoms, and those formed in ethanol/water containing 64 atoms. The largest cluster used for the refinement is approx. 13.5 × 8.2 × 6.5 $Å^3$ along the hexagonal crystallographic axes. The intermediate clusters are approx. 3.5 × 5.5 ×

6.5 Å^3 and the smallest cluster is 8.1 × 8.1 × 6.5 Å^3. Several hcp clusters 0.3–0.6 Å larger in different directions provided fits of almost the same quality, which indicates a distributed size of the NPs consistent with the SAXS and TEM data. Examples of fits using other types of clusters are seen in Figures S28–S31.

The sizes of the clusters used for the refinement should not be taken as absolute sizes of the NPs themselves, but instead describe any anisotropy induced from the hcp structure on the NP shape. To verify the validity of the cluster models, we also fitted the PDFs with spherical NPs with a single particle diameter and a lognormal size distribution, and those fits are seen in Figures S32–S38. For the smallest NPs, formed from $OsCl_3$ in ethanol/water, all models give a fit of the same quality, which is reasonable as the cluster model identified (8.1 × 8.1 × 6.5 Å^3) is almost isotropic. However, for the largest NPs formed from H_2OsCl_6 in methanol/water, the discrete, anisotropic cluster model provides a better fit than that from a spherical particle model. This illustrates that for an accurate description of the atomic structure of small NPs it is important to consider any possible anisotropy induced by the crystal structure.

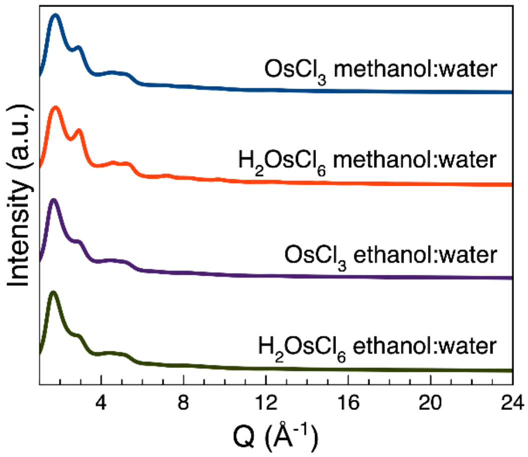

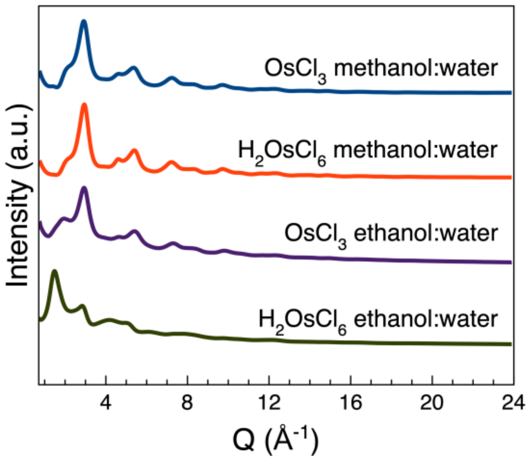

Figure S25: Total scattering data before (left) and after (right) background subtraction.

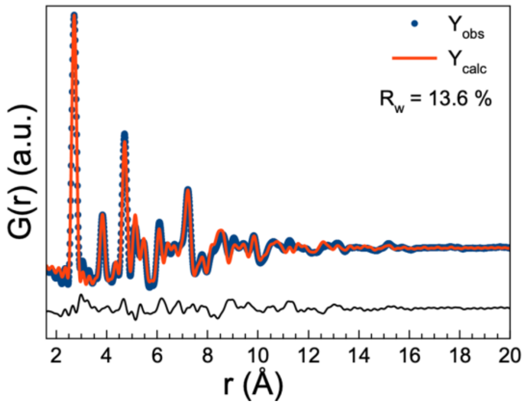

Figure S26: Fit using an hcp cluster from ASE to the PDF obtained from the NPs formed from H_2OsCl_6 in methanol/water. See refinement parameters in Table S4.

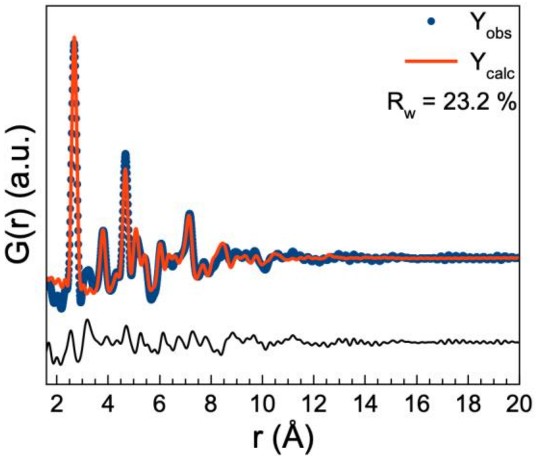

Figure S27: Fit using an hcp cluster from ASE to the PDF obtained from the NPs formed from $OsCl_3$ in ethanol/water. See refinement parameters in Table S5.

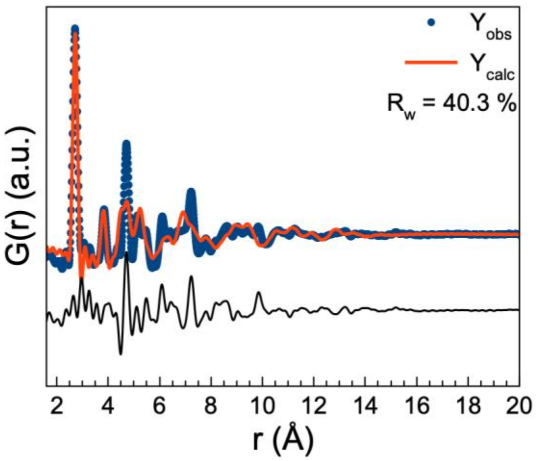

Figure S28: Fit using an icosahedral cluster from ASE to the PDF obtained from the NPs formed from $OsCl_3$ in methanol/water.

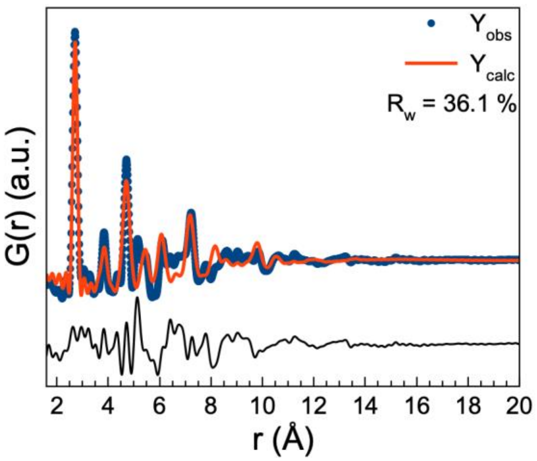

Figure S29: Fit using an octahedral cluster from ASE to the PDF obtained from the NPs formed from $OsCl_3$ in methanol/water.

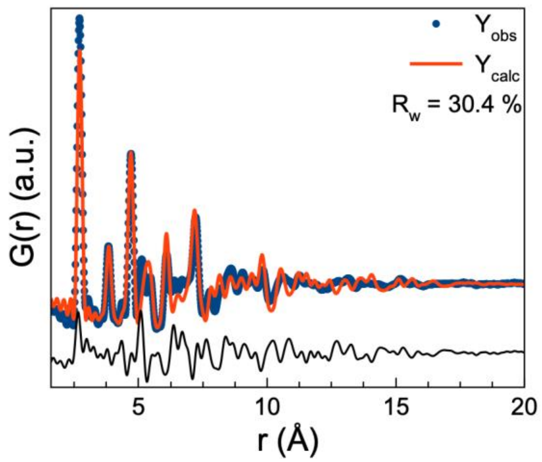

Figure S30: Fit using a decahedral cluster from ASE to the PDF obtained from the NPs formed from H_2OsCl_6 in methanol/water.

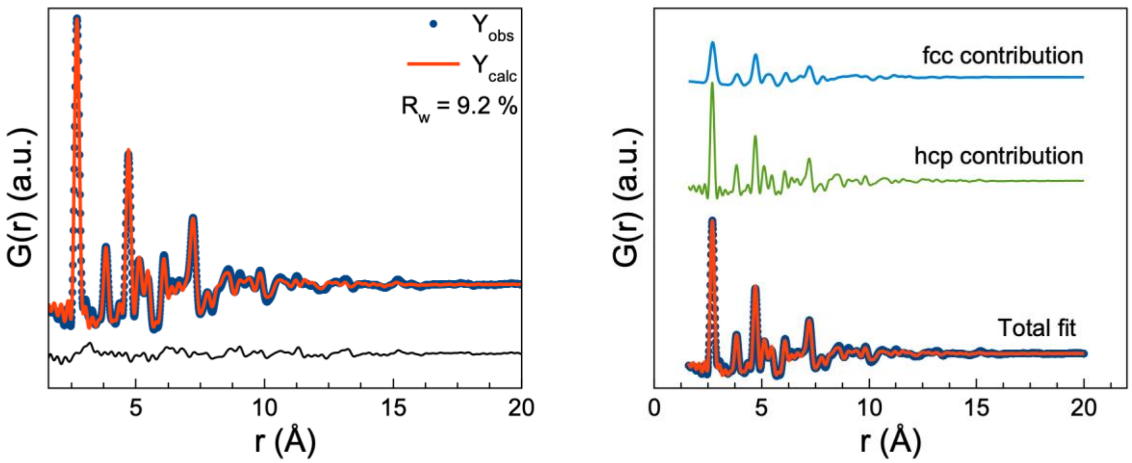

Figure S31: Fit improvement with two-phase models using an hcp cluster and decahedron for PDF obtained from the nanoparticles formed from H_2OsCl_6 in methanol/water.

The fits of can be further improved by fitting with a combination of an hcp cluster and a decahedron cluster. On the left is the total fit and on the right is the fit including the

individual contributions, with 64 weight % hcp and 36 weight % decahedral. The hcp and fcc structures in the clusters are very similar and it is likely that the improvement simply comes from adding more parameters to the fit. The improvement to the fit could represent the presence of Os NPs with a decahedron structure, but at the moment we cannot absolutely confirm this.

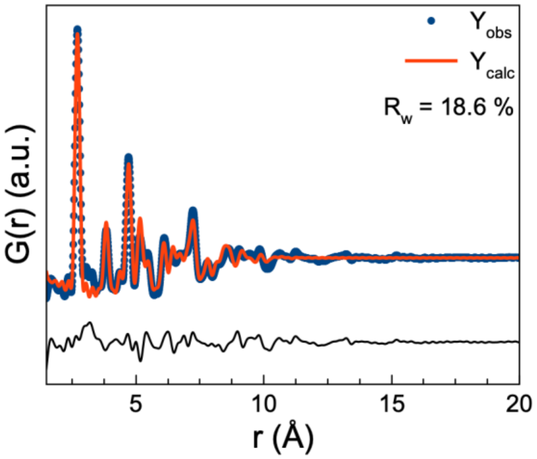

Figure S32: Fit using a spherical hcp particle to the PDF obtained from the NPs formed from $OsCl_3$ in methanol/water. See refinement parameters in Table S6.

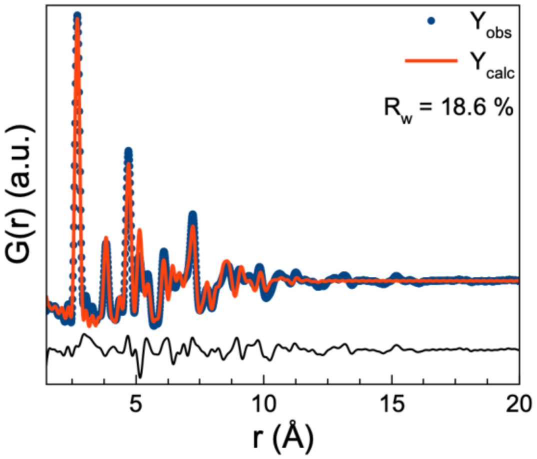

Figure S33: Fit using a spherical hcp particle to the PDF obtained from the NPs formed from H_2OsCl_6 in methanol/water. See refinement parameters in Table S7.

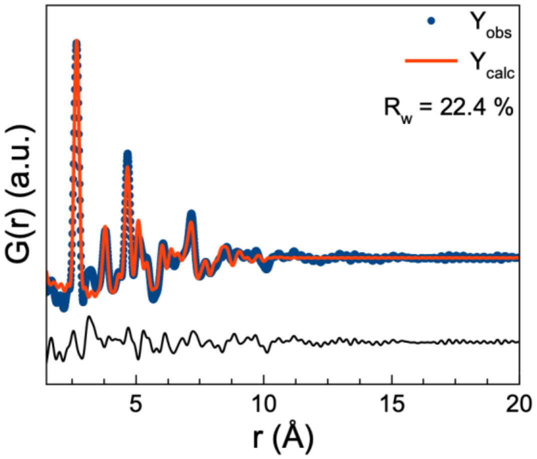

Figure S34: Fit using a spherical hcp particle to the PDF obtained from the NPs formed from $OsCl_3$ in ethanol/water. See refinement parameters in Table S8.

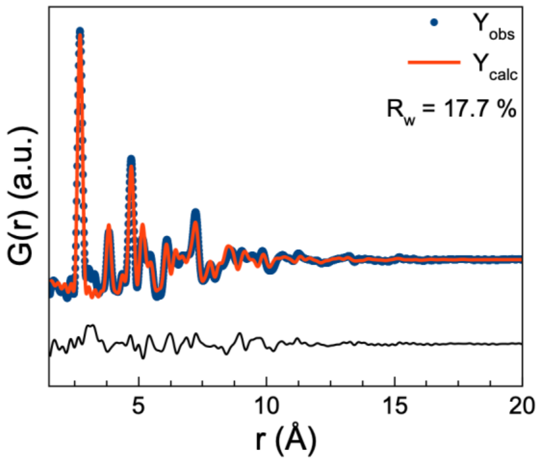

Figure S35: Fit using a spherical hcp particle shape with a lognormal distribution to the PDF obtained from the NPs formed from $OsCl_3$ in methanol/water. See refinement parameters in Table S9 and the refined distribution in Figure S38.

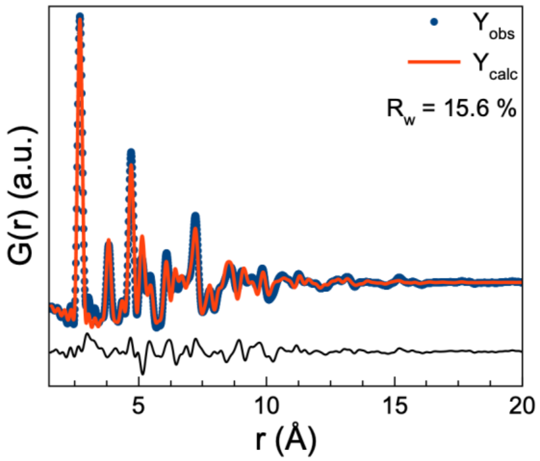

Figure S36: Fit using a spherical hcp particle shape with a lognormal distribution to the PDF obtained from the NPs formed from H_2OsCl_6 in methanol/water. See refinement parameters in Table S10 and the refined distribution in Figure S38.

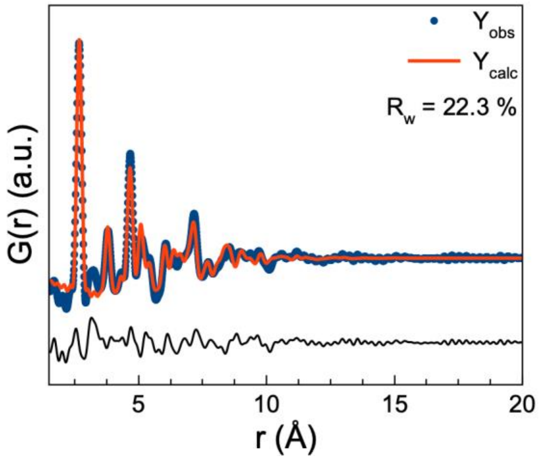

Figure S37: Fit using a spherical hcp particle shape with a lognormal distribution to the PDF obtained from the NPs formed from $OsCl_3$ in ethanol/water. See refinement parameters in Table S11 and the refined distribution in Figure S38.

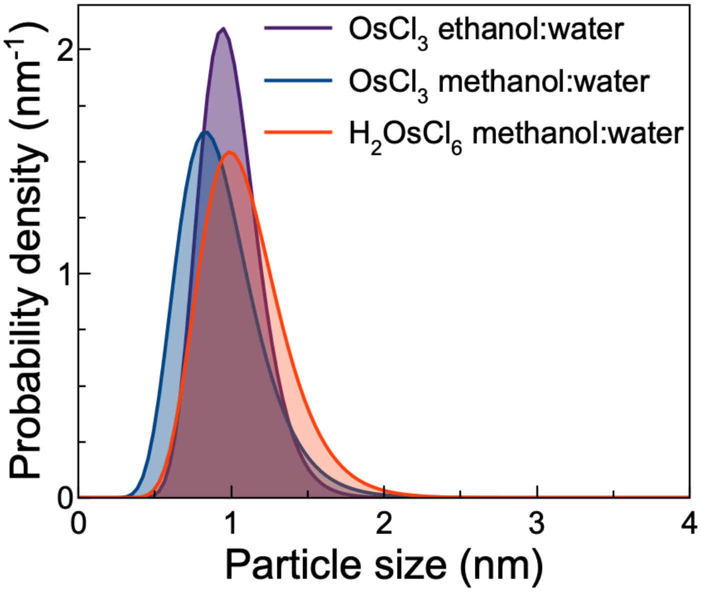

Figure S38: The lognormal size distributions obtained from the PDF fits seen in Figures S35–S37. The size distribution obtained from the PDF agrees with the ones obtained by SAXS.

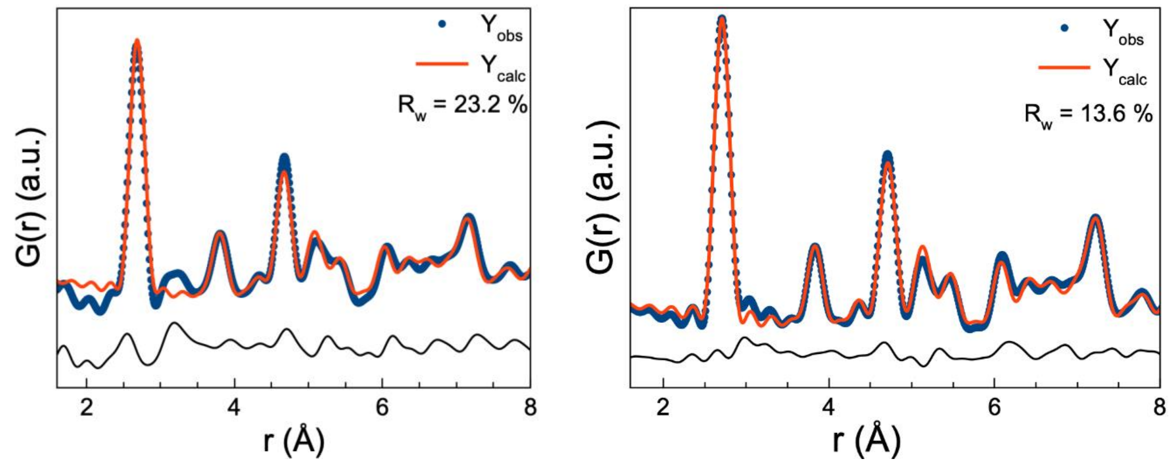

Figure S39: Zoom on the fits seen in Figure S27 (left) and Figure S26 (right). An impurity is visible as a small peak at 3.2 Å. The possible Os–Cl peak at 2.3 Å is mostly visible on the left and is a small shoulder to the main Os–Os peak at 2.7 Å.

As mentioned in the main text, the samples contain an impurity giving rise to a peak at 3.2 Å. The only known distance that could explain the observation of a distance at 3.2 Å is the Os–Os distance in a Os_2Cl_2 complex, for example, seen in $Os_3Cl_2(CO)_{10}$ [16]. The presence of a Os_2Cl_2 complex would also give a Os–Cl peak at 2.3 Å, which might be present as a shoulder on the main Os–Os peak at 2.7 Å in the experimental PDF, as seen in Figure S39. $[Os_xCl_y(CO)_z]$ might form here since, by analogy with Pt NP formation [17], it can be expected that some CO form by oxidation of the alcohols during reduction of Os.

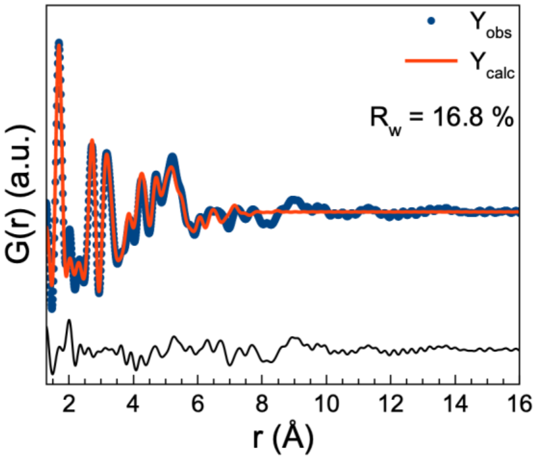

Figure S40: Fit using a combination of hcp Os and OsO_4 to the PDF obtained from the NPs formed from H_2OsCl_6 in ethanol/water. See refinement parameters in Tables S6 and S7.

The peak at 1.7 Å corresponds well to the Os–O distances in OsO_4 and the PDF obtained from these NPs can be fitted by a combination of hcp Os and OsO_4. We attribute this different behaviour to the likely oxidation of the sample at the time of measurement and the sample contained 97.5 wt % OsO_4 and 2.5 wt % Os. The sample oxidation is likely due to unintended differences in the storage or shipping conditions and show that while the NPs can remain un-oxidised in solutions in sealed containers, they are likely to oxidise over time or after solvent removal. The refined average diameter of the Os NPs was found to be 0.95 nm and the OsO_4 NP show an average diameter of 0.85 nm. However, the difference in the curve from the fit shows that slightly larger particles are also present as the PDF peaks extends to 1.3 nm.

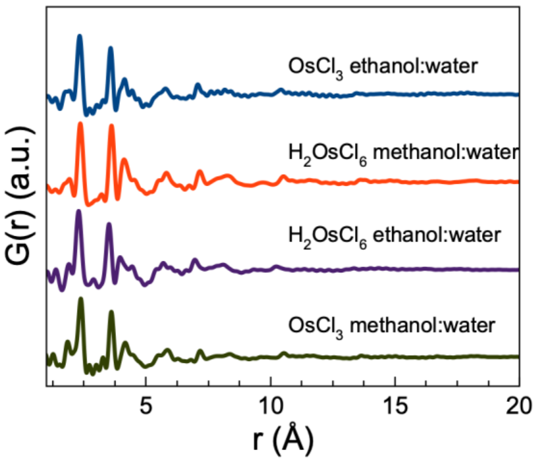

Figure S41: The PDFs obtained from the initial precursor at room temperature. The PDFs for each solution are almost identical and, as elaborated further in Figures S42–S45, the two first distances are at 1.8 and 2.3 Å, which corresponds to Os–O and Os–Cl bonds, respectively.

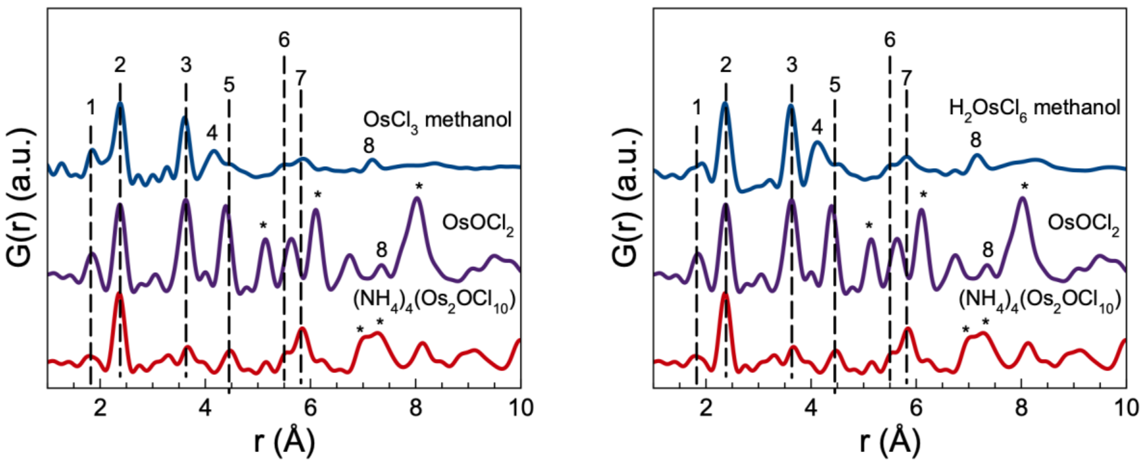

Figure S42: Comparison of the precursor PDFs to the calculated PDFs of $OsOCl_2$ and $(NH_4)_4(OsOCl_{10})$ with the most important distances indicated and with nitrogen distances omitted. Osmium, oxygen, and chlorine are known to form chain-like structures with Os octahedrally coordinated to O and Cl, two examples are $OsOCl_2$ [18] and $(NH_4)_4(OsOCl_{10})$ [19] whose crystal structures can be seen in Figures S43 and S44. The eight distances are indicated on Figure S45.

Distances 1 and 2 are the direct bond distances between Os–O and Os–Cl. Distance 3 is the distance between two Os atoms bridged either by a single O atom or two Cl atoms. The fourth distance is not present in the reported crystal structures but is directly related to distance 5. As indicated in Figure S37, distance 5 is an Os–Cl distance. If the Cl atom is replaced with an O atom and the O is moved such that the Os–O bond (distance 1) becomes 1.8 Å, distance 5 is shortened from 4.5 Å to 4.2 Å which is the length of distance 4, as shown in Figure S39B. This type of Os–O distance is not present in the original structures, which indicates a disordered placement of Cl and O in the octahedral coordination. As indicated in Figure S45, distance 6 and 7 are Os–O and Os–Cl distances, respectively. Distance 8 is 7.2 Å long which is exactly twice the value of distance 3, which is 3.6 Å. In $OsOCl_2$ the Os–Os distance in distance 3 is 3.7 Å and therefore the distance 8 peak moved to the right, up to 7.4 Å. The distances indicated with stars are distances that corresponds to the interatomic distances between the chains in the crystal structures. From this analysis we can conclude that the Os precursors react with the alcohol and form chain-like structures of [$OsCl_xO_y$]-octahedra. The chains are roughly 1–1.5 nm long and are likely kept together by both O and Cl bridges.

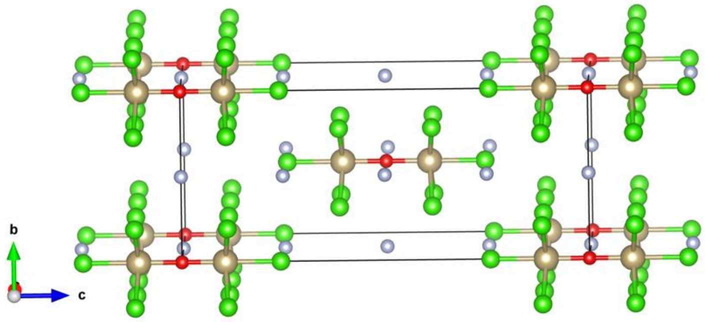

Figure S43: The crystal structure of $(NH_4)_4(OsOCl_{10})$ viewed along a. Os is shown in grey, O in red, Cl in green; and N in blue. H is omitted for clarity.

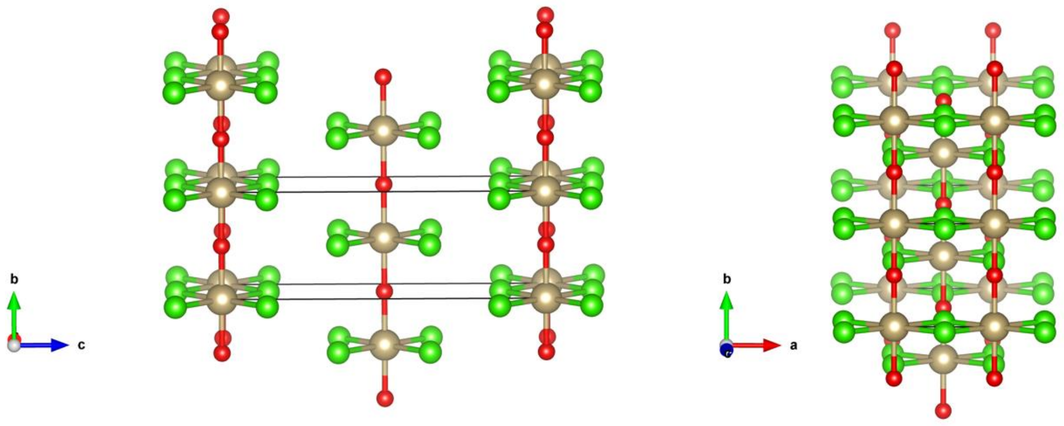

Figure S44: The crystal structure of $OsOCl_2$ viewed along a and c. Os is shown in grey, O in red, and Cl in green.

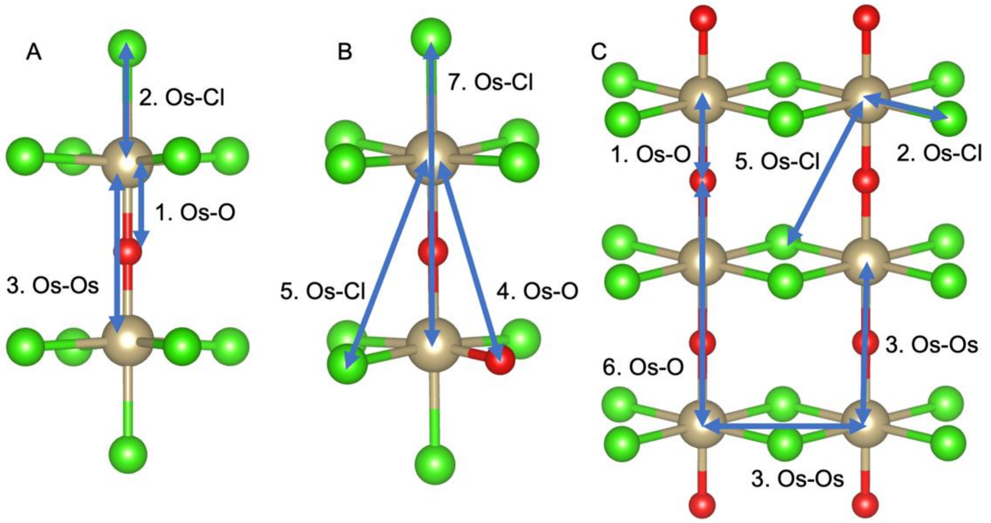

Figure S45: The visualization of the distances discussed in Figure S42. (A) shows the original $[OsOCl_{10}]^{4-}$ complex from $(NH_4)_4(OsOCl_{10})$ and (B) shows the same complex but with one Cl replaced by a O to give distance 4. (C) shows part of the $OsOCl_2$ structure.

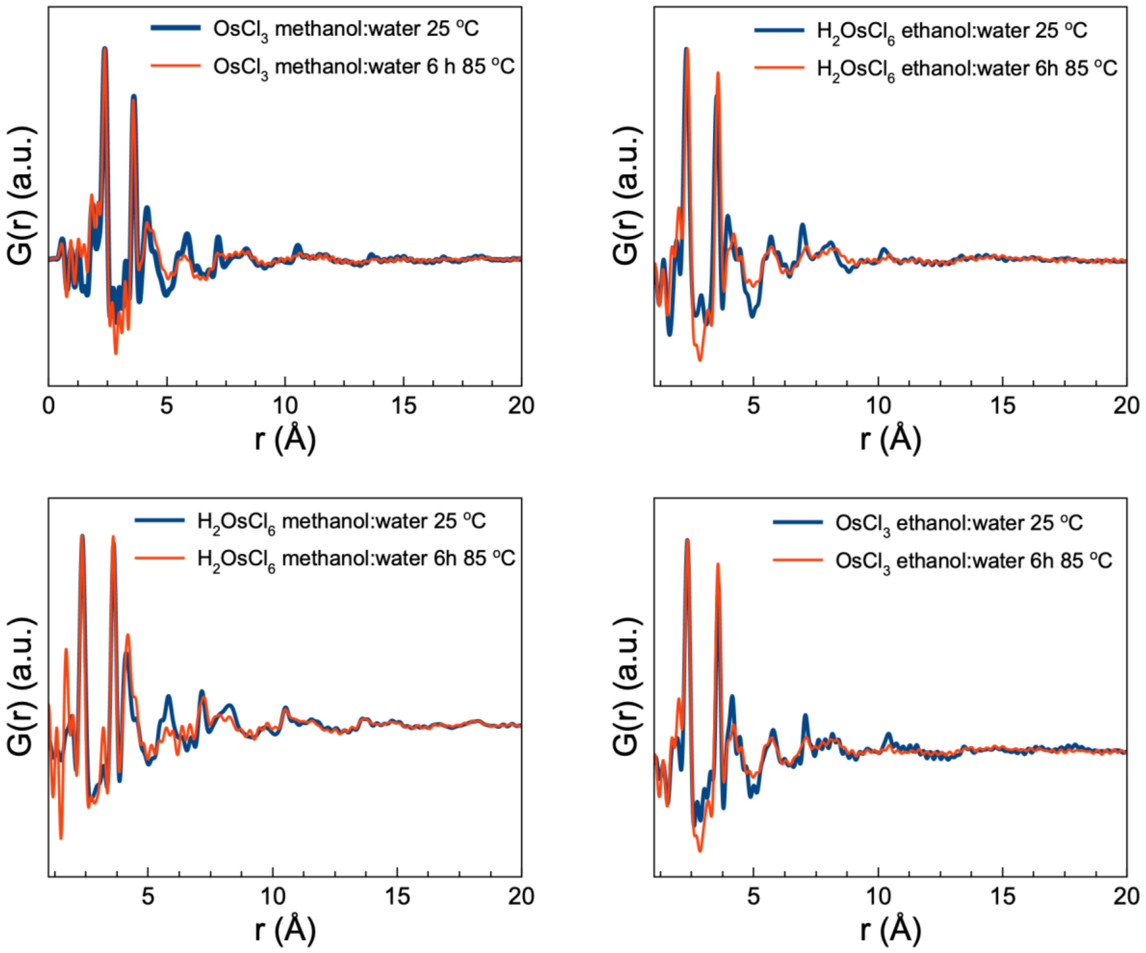

Figure S46: The PDFs of the precursors at room temperature and after being heated at 85 °C for 6 h in a 3 mm NMR tube at a precursor concentration of 100 mM. The only real change is the loss of intensity at high *r*, which means that the chain-like structures are breaking apart and getting smaller. However, the local coordination remains almost unchanged, exempt from an increase in intensity for the Os–O distance at 1.8 Å. The increase in intensity suggests a replacement of Cl with O.

Table S3: Refined parameters for the fit in Figure 3, including the atomic positions in the hcp cluster.

δ_2	2.04 Å^2	U_{iso}	0.004 Å^2
**Atom**	**x**	**y**	**z**
Os	0.0	0.0	0.0
Os	0.0	1.578605	2.177253
Os	2.734224	0.0	0.0
Os	2.734224	1.578605	2.177253
Os	5.468449	0.0	0.0
Os	5.468449	1.578605	2.177253
Os	-1.367112	2.367908	0.0
Os	-1.367112	3.946513	2.177253
Os	1.367112	2.367908	0.0
Os	1.367112	3.946513	2.177253
Os	4.101337	2.367908	0.0
Os	4.101337	3.946513	2.177253
Os	-2.734224	4.735815	0.0
Os	-2.734224	6.314421	2.177253
Os	0.0	4.735815	0.0
Os	0.0	6.314421	2.177253
Os	2.734224	4.735815	0.0
Os	2.734224	6.314421	2.177253
Os	-4.101337	7.103724	0.0
Os	-4.101337	8.682328	2.177253
Os	-1.367112	7.103724	0.0
Os	-1.367112	8.682328	2.177253
Os	1.367112	7.103724	0.0
Os	1.367112	8.682328	2.177253
Os	-5.468449	9.471631	0.0
Os	-5.468449	11.050236	2.177253
Os	-2.734224	9.471631	0.0
Os	-2.734224	11.050236	2.177253
Os	0.0	9.471631	0.0
Os	0.0	11.050236	2.177253
Os	-6.835561	11.839539	0.0
Os	-6.835561	13.418145	2.177253
Os	-4.101337	11.839539	0.0
Os	-4.101337	13.418145	2.177253
Os	-1.367112	11.839539	0.0
Os	-1.367112	13.418145	2.177253
Os	0.0	0.0	4.354506
Os	0.0	1.578605	6.531758
Os	2.734224	0.0	4.354506
Os	2.734224	1.578605	6.531758
Os	5.468449	0.0	4.354506
Os	5.468449	1.578605	6.531758
Os	-1.367112	2.367908	4.354506
Os	-1.367112	3.946513	6.531758
Os	1.367112	2.367908	4.354506
Os	1.367112	3.946513	6.531758
Os	4.101337	2.367908	4.354506
Os	4.101337	3.946513	6.531758

Os	-2.734224	4.735815	4.354506
Os	-2.734224	6.314421	6.531758
Os	0.0	4.735815	4.354506
Os	0.0	6.314421	6.531758
Os	2.734224	4.735815	4.354506
Os	2.734224	6.314421	6.531758
Os	-4.101337	7.103724	4.354506
Os	-4.101337	8.682328	6.531758
Os	-1.367112	7.103724	4.354506
Os	-1.367112	8.682328	6.531758
Os	1.367112	7.103724	4.354506
Os	1.367112	8.682328	6.531758
Os	-5.468449	9.471631	4.354506
Os	-5.468449	11.050236	6.531758
Os	-2.734224	9.471631	4.354506
Os	-2.734224	11.050236	6.531758
Os	0.0	9.471631	4.354506
Os	0.0	11.050236	6.531758
Os	-6.835561	11.839539	4.354506
Os	-6.835561	13.418145	6.531758
Os	-4.101337	11.839539	4.354506
Os	-4.101337	13.418145	6.531758
Os	-1.367112	11.839539	4.354506
Os	-1.367112	13.418145	6.531758

Table S4: Refined parameters for the fit in Figure S26, including the atomic positions in the hcp cluster.

δ_2	2.13 Å^2	U_{iso}	0.004 Å^2
**Atom**	**x**	**y**	**x**
Os	0.0	0.0	0.0
Os	0.0	1.576459	2.174293
Os	2.730507	0.0	0.0
Os	2.730507	1.576459	2.174293
Os	5.461015	0.0	0.0
Os	5.461015	1.576459	2.174293
Os	8.191522	0.0	0.0
Os	8.191522	1.576459	2.174293
Os	-1.365254	2.364689	0.0
Os	-1.365254	3.941148	2.174293
Os	1.365254	2.364689	0.0
Os	1.365254	3.941148	2.174293
Os	4.095761	2.364689	0.0
Os	4.095761	3.941148	2.174293
Os	6.826268	2.364689	0.0
Os	6.826268	3.941148	2.174293
Os	-2.730507	4.729377	0.0
Os	-2.730507	6.305837	2.174293
Os	0.0	4.729377	0.0
Os	0.0	6.305837	2.174293
Os	2.730507	4.729377	0.0
Os	2.730507	6.305837	2.174293
Os	5.461015	4.729377	0.0
Os	5.461015	6.305837	2.174293

Os	-4.095761	7.094066	0.0
Os	-4.095761	8.670525	2.174293
Os	-1.365254	7.094066	0.0
Os	-1.365254	8.670525	2.174293
Os	1.365254	7.094066	0.0
Os	1.365254	8.670525	2.174293
Os	4.095761	7.094066	0.0
Os	4.095761	8.670525	2.174293
Os	-5.461015	9.458755	0.0
Os	-5.461015	11.035214	2.174293
Os	-2.730507	9.458755	0.0
Os	-2.730507	11.035214	2.174293
Os	0.0	9.458755	0.0
Os	0.0	11.035214	2.174293
Os	2.730507	9.458755	0.0
Os	2.730507	11.035214	2.174293
Os	-6.826268	11.823444	0.0
Os	-6.826268	13.399903	2.174293
Os	-4.095761	11.823444	0.0
Os	-4.095761	13.399903	2.174293
Os	-1.365254	11.823444	0.0
Os	-1.365254	13.399903	2.174293
Os	1.365254	11.823444	0.0
Os	1.365254	13.399903	2.174293
Os	0.0	0.0	4.348586
Os	0.0	1.576459	6.522879
Os	2.730507	0.0	4.348586
Os	2.730507	1.576459	6.522879
Os	5.461015	0.0	4.348586
Os	5.461015	1.576459	6.522879
Os	8.191522	0.0	4.348586
Os	8.191522	1.576459	6.522879
Os	-1.365254	2.364689	4.348586
Os	-1.365254	3.941148	6.522879
Os	1.365254	2.364689	4.348586
Os	1.365254	3.941148	6.522879
Os	4.095761	2.364689	4.348586
Os	4.095761	3.941148	6.522879
Os	6.826268	2.364689	4.348586
Os	6.826268	3.941148	6.522879
Os	-2.730507	4.729377	4.348586
Os	-2.730507	6.305837	6.522879
Os	0.0	4.729377	4.348586
Os	0.0	6.305837	6.522879
Os	2.730507	4.729377	4.348586
Os	2.730507	6.305837	6.522879
Os	5.461015	4.729377	4.348586
Os	5.461015	6.305837	6.522879
Os	-4.095761	7.094066	4.348586
Os	-4.095761	8.670525	6.522879
Os	-1.365254	7.094066	4.348586
Os	-1.365254	8.670525	6.522879
Os	1.365254	7.094066	4.348586
Os	1.365254	8.670525	6.522879
Os	4.095761	7.094066	4.348586
Os	4.095761	8.670525	6.522879
Os	-5.461015	9.458755	4.348586
Os	-5.461015	11.035214	6.522879

Os	-2.730507	9.458755	4.348586
Os	-2.730507	11.035214	6.522879
Os	0.0	9.458755	4.348586
Os	0.0	11.035214	6.522879
Os	2.730507	9.458755	4.348586
Os	2.730507	11.035214	6.522879
Os	-6.826268	11.823444	4.348586
Os	-6.826268	13.399903	6.522879
Os	-4.095761	11.823444	4.348586
Os	-4.095761	13.399903	6.522879
Os	-1.365254	11.823444	4.348586
Os	-1.365254	13.399903	6.522879
Os	1.365254	11.823444	4.348586
Os	1.365254	13.399903	6.522879

Table S5: Refined parameters for the fit in Figure S27, including the atomic positions in the hcp cluster.

δ_2	2.11 Å^2	U_{iso}	0.005 Å^2
Atom	x	y	z
Os	0.0	0.0	0.0
Os	0.0	1.561427	2.15356
Os	2.704471	0.0	0.0
Os	2.704471	1.561427	2.15356
Os	5.408942	0.0	0.0
Os	5.408942	1.561427	2.15356
Os	8.113414	0.0	0.0
Os	8.113414	1.561427	2.15356
Os	-1.352236	2.342141	0.0
Os	-1.352236	3.903568	2.15356
Os	1.352236	2.342141	0.0
Os	1.352236	3.903568	2.15356
Os	4.056707	2.342141	0.0
Os	4.056707	3.903568	2.15356
Os	6.761178	2.342141	0.0
Os	6.761178	3.903568	2.15356
Os	-2.704471	4.684281	0.0
Os	-2.704471	6.245709	2.15356
Os	0.0	4.684281	0.0
Os	0.0	6.245709	2.15356
Os	2.704471	4.684281	0.0
Os	2.704471	6.245709	2.15356
Os	5.408942	4.684281	0.0
Os	5.408942	6.245709	2.15356
Os	-4.056707	7.026422	0.0
Os	-4.056707	8.587849	2.15356
Os	-1.352236	7.026422	0.0
Os	-1.352236	8.587849	2.15356
Os	1.352236	7.026422	0.0
Os	1.352236	8.587849	2.15356
Os	4.056707	7.026422	0.0
Os	4.056707	8.587849	2.15356
Os	0.0	0.0	4.307121
Os	0.0	1.561427	6.460681

Os	2.704471	0.0	4.307121
Os	2.704471	1.561427	6.460681
Os	5.408942	0.0	4.307121
Os	5.408942	1.561427	6.460681
Os	8.113414	0.0	4.307121
Os	8.113414	1.561427	6.460681
Os	-1.352236	2.342141	4.307121
Os	-1.352236	3.903568	6.460681
Os	1.352236	2.342141	4.307121
Os	1.352236	3.903568	6.460681
Os	4.056707	2.342141	4.307121
Os	4.056707	3.903568	6.460681
Os	6.761178	2.342141	4.307121
Os	6.761178	3.903568	6.460681
Os	-2.704471	4.684281	4.307121
Os	-2.704471	6.245709	6.460681
Os	0.0	4.684281	4.307121
Os	0.0	6.245709	6.460681
Os	2.704471	4.684281	4.307121
Os	2.704471	6.245709	6.460681
Os	5.408942	4.684281	4.307121
Os	5.408942	6.245709	6.460681
Os	-4.056707	7.026422	4.307121
Os	-4.056707	8.587849	6.460681
Os	-1.352236	7.026422	4.307121
Os	-1.352236	8.587849	6.460681
Os	1.352236	7.026422	4.307121
Os	1.352236	8.587849	6.460681
Os	4.056707	7.026422	4.307121
Os	4.056707	8.587849	6.460681

Table S6: Refined parameters for Os in the fit shown in Figure S32.

	**Os**	**Space group**	**P 6/3 mmc**		
**a**	**c**	**Spherical diameter**	**δ_2**		
2.73 Å	4.38 Å	12.04 Å	2.07 Å^2		
**Atoms**	**x**	**y**	**z**	**U_{iso}**	
**Os**	1/3	2/3	0.25	0.004 Å^2	

Table S7: Refined parameters for Os in the fit shown in Figure S33.

	**Os**	**Space group**	**P 6/3 mmc**		
**a**	**c**	**Spherical diameter**	**δ_2**		
2.73 Å	4.36 Å	13.15 Å	2.08 Å^2		
**Atoms**	**x**	**y**	**z**	**U_{iso}**	
**Os**	1/3	2/3	0.25	0.004 Å^2	

Table S8: Refined parameters for Os in the fit shown in Figure S34.

	**Os**	**Space group**	**P 6/3 mmc**		
**a**	**c**	**Spherical diameter**	**δ_2**		
2.71 Å	4.32 Å	11.60 Å	1.46 Å		
**Atoms**	**x**	**y**	**z**	**U_{iso}**	
**Os**	1/3	2/3	0.25	0.004 Å^2	

Table S9: Refined parameters for Os in the fit shown in Figure S35.

	**Os**	**Space group**	**P 6/3 mmc**		
**a**	**c**	**Spherical diameter**	**Size deviation**	**δ_2**	
2.73 Å	4.38 Å	9.36 Å	2.71	1.94 Å^2	
**Atoms**	**x**	**y**	**z**	**U_{iso}**	
**Os**	1/3	2/3	0.25	0.005 Å^2	

Table S10: Refined parameters for Os in the fit shown in Figure S36.

	Os	Space group	P 6/3 mmc		
**a**	**c**	**Spherical diameter**	**Size deviation**	**δ_2**	
2.73 Å	4.36 Å	10.09 Å	2.82 Å^2	2.08 Å^2	
**Atoms**	**x**	**y**	**z**	**U_{iso}**	
**Os**	1/3	2/3	0.25	0.005 Å^2	

Table S11: Refined parameters for Os in the fit shown in Figure S37.

	Os	Space group	P 6/3 mmc		
**a**	**c**	**Spherical diameter**	**Size deviation**	**δ_2**	
2.71 Å	4.32 Å	10.05 Å	1.97	1.46 Å^2	
**Atoms**	**x**	**y**	**z**	**U_{iso}**	
**Os**	1/3	2/3	0.25	0.006 Å^2	

Table S12: Refined parameters for OsO_4 in the fit shown in Figure S40.

	OsO_4	Space group	C 2/c		
**a**	**b**	**c**	**β**	**Spherical diameter**	**δ_2**
9.18 Å	4.21 Å	9.85 Å	127.364°	8.55 Å	1.9 Å^2
**Atoms**	**x**	**y**	**z**	**U_{iso}**	
**Os**	0	0.16	0.25	0.005 Å^2	
**O1**	0.14	0.0007	0.20	0.006 Å^2	
**O2**	0.12	0.44	0.11	0.006 Å^2	

Table S13: Refined parameters for Os in the fit shown in Figure S40.

	Os	Space group	P 6/3 mmc		
**a**	**c**	**Spherical diameter**	δ_2		
2.71 Å	4.45 Å	9.38 Å	1.46 Å^2		
**Atoms**	**x**	**y**	**z**	U_{iso}	
**Os**	1/3	2/3	0.25	0.002 Å^2	

References

1. Chupas, P. J.; Qiu, X.; Hanson, J. C.; Lee, P. L.; Grey, C. P.; Billinge, S. J. L. *J. Appl. Crystallogr.* **2003**, *36* (6), 1342-1347.

2. Hammersley, A. *J. Appl. Crystallogr.* **2016**, *49* (2), 646-652.

3. Prescher, C.; Prakapenka, V. B. *High Press Res* **2015**, *35* (3), 223-230.

4. X. J. Yang, Pavol; Farrow, Christopher L.; Billinge, Simon J. L., **2014**. arXiv:1402.3163v3. https://arxiv.org/abs/1402.3163.

5. Farrow, C. L.; Juhas, P.; Liu, J. W.; Bryndin, D.; Božin, E. S.; Bloch, J.; Th, P.; Billinge, S. J. L. *J. Phys.: Condens. Matter* **2007**, *19* (33), 335219.

6. Juhas, P.; Farrow, C. L.; Yang, X.; Knox, K. R.; Billinge, S. J. L. *Acta Cryst. A* **2015**, *71* (6), 562-568.

7. Hjorth Larsen, A.; Jørgen Mortensen, J.; Blomqvist, J.; Castelli, I. E.; Christensen, R.; Dułak, M.; Friis, J.; Groves, M. N.; Hammer, B.; Hargus, C.; Hermes, E. D.; Jennings, P. C.; Bjerre Jensen, P.; Kermode, J.; Kitchin, J. R.; Leonhard Kolsbjerg, E.; Kubal, J.;

Kaasbjerg, K.; Lysgaard, S.; Bergmann Maronsson, J.; Maxson, T.; Olsen, T.; Pastewka, L.; Peterson, A.; Rostgaard, C.; Schiøtz, J.; Schütt, O.; Strange, M.; Thygesen, K. S.; Vegge, T.; Vilhelmsen, L.; Walter, M.; Zeng, Z.; Jacobsen, K. W. *J. Phys.: Condens. Matter.* **2017**, *29* (27), 273002.

8. Jensen, K. M. O.; Juhas, P.; Tofanelli, M. A.; Heinecke, C. L.; Vaughan, G.; Ackerson, C. J.; Billinge, S. J. L. *Nat. Commun.* **2016**, *7*, ID11859

9. Schroder, J.; Quinson, J.; Mathiesen, J. K.; Kirkensgaard, J. J. K.; Alinejad, S.; Mints, V. A.; Jensen, K. M. O.; Arenz, M. *J. Electrochem. Soc.* **2020**, *167* (13).

10. T. Zemb and P. Lindner, *Neutrons, X-rays and light: scattering methods applied to soft condensed matter*, Elsevier, **2002**.

11. Garcia, P.; Prymak, O.; Grasmik, V.; Pappert, K.; Wlysses, W.; Otubo, L.; Epple, M.; Oliveira, C. L. P. *Nanoscale Adv.* **2020**, *2* (1), 225-238.

12. Wakisaka, T.; Kusada, K.; Yamamoto, T.; Toriyama, T.; Matsumura, S.; Ibrahima, G.; Seo, O.; Kim, J.; Hiroi, S.; Sakata, O.; Kawaguchi, S.; Kubota, Y.; Kitagawa, H. *Chem. Commun.* **2020**, *56* (3), 372-374.

13. Wang, Y.; Zhang, J. L.; Wang, X. D.; Ren, J. W.; Zuo, B. J.; Tang, Y. Q. *Top. Catal.* **2005**, *35* (1-2), 35-41.

14. Hirai, H.; Nakao, Y.; Toshima, N. *J. Macromol. Sci. Chem.* **1979**, *A13* (6), 727-750.

15. He, S. B.; Yang, L.; Balasubramanian, P.; Li, S. J.; Peng, H. P.; Kuang, Y.; Deng, H. H.; Chen, W. *J. of Mater. Chem. A* **2020**, *8* (47), 25226-25234.

16. Einstein, F. W. B.; Jones, T.; Tyers, K. G. *Acta Crystallogr. B* **1982**, *38* (4), 1272-1274.

17. Quinson, J.; Neumann, S.; Kacenauskaite, L.; Bucher, J.; Kirkensgaard, J. J. K.; Simonsen, S. B.; Kuhn, L. T.; Zana, A.; Vosch, T.; Oezaslan, M.; Kunz, S.; Arenz, M. *Chem. Eur. J.* **2020**, *26* (41), 9012-9023.

18. Hillebrecht, H.; Schmidt, P. J.; Rotter, H. W.; Thiele, G.; Zönnchen, P.; Bengel, H.; Cantow, H. J.; Magonov, S. N.; Whangbo, M. H. *J. Alloys Compd.* **1997**, *246* (1), 70-79.

19. Rudnitskaya, O. V.; Kultyshkina, E. K.; Popova, M. S.; Stash, A. I. *Russ. J. Inorg. Chem.* **2013**, *58* (10), 1227-1230.
